# Effects of river regulation on aquatic invertebrate community composition: A comparative analysis in two southern African rivers

**DOI:** 10.1002/ece3.10963

**Published:** 2024-02-07

**Authors:** Lizaan de Necker, Divan van Rooyen, Ruan Gerber, Luc Brendonck, Victor Wepener, Nico J. Smit

**Affiliations:** ^1^ Water Research Group, Unit for Environmental Sciences and Management North‐West University Potchefstroom South Africa; ^2^ South African Institute for Aquatic Biodiversity (NRF‐SAIAB) Makhanda South Africa; ^3^ Animal Ecology, Global Change and Sustainable Development, Department of Biology University of Leuven Leuven Belgium

**Keywords:** community composition, conservation, drought, floodplain, functional diversity, invasive species, natural ecosystem, river regulation, water quality

## Abstract

While natural floods play a crucial role in shaping the composition of aquatic communities, the most rivers worldwide are regulated or dammed for anthropogenic purposes, resulting in alterations to the biological and chemical composition of these ecosystems. Studies have demonstrated various negative effects of river regulation on aquatic invertebrate communities globally. However, there is a scarcity of research in Africa, despite its vulnerability to anthropogenic impacts. This study aimed to compare aquatic invertebrate communities in the Phongolo River, an impacted regulated river, and the Usuthu River, a less impacted unregulated river, in South Africa. It further aimed to ascertain whether Lake Nyamithi, a naturally saline lake receiving water from both of the aforementioned systems, exhibited a stronger similarity to one of the two rivers in terms of its aquatic invertebrate composition. Aquatic invertebrate and water samples were collected from 2012 to 2018 over several surveys. The Usuthu River demonstrated a diverse and sensitive aquatic invertebrate community, emphasising its high conservation value. The Phongolo River demonstrated effects of anthropogenic impact, with taxa more resilient to changes in water quality and flow compared to the Usuthu River. Mismanagement and excessive water use may lead to the loss of any remaining sensitive aquatic invertebrate communities in this river. The presence of invasive molluscan in the Phongolo River and Lake Nyamithi also poses a threat to the native aquatic invertebrate communities. These invasive species are currently absent from the Usuthu River although other invasive species, such as the Australian redclaw crayfish, are found in both river systems. Lake Nyamithi displayed a unique aquatic invertebrate community, distinct from both rivers and their floodplains. This study provides important baseline information on the Usuthu River's aquatic invertebrates and emphasises the need to maintain adequate water flow in rivers and floodplains to protect biodiversity and sensitive species.

## Graphical abstract

This study compared aquatic invertebrate communities between a regulated and unregulated river and sought to determine whether the largest inland lake of the region, which receives water from both rivers, resembles the aquatic invertebrate communities of either river. The unregulated river showed a diverse and sensitive aquatic invertebrate community, indicating its high conservation value, while the regulated river has experienced effects of anthropogenic impacts, with a greater presence of invertebrates resilient to changes in water quality. The inland lake displayed a unique aquatic invertebrate community, different from both rivers and their floodplains. This study underscores the importance of maintaining adequate water flow in rivers and floodplains to protect biodiversity and sensitive species.

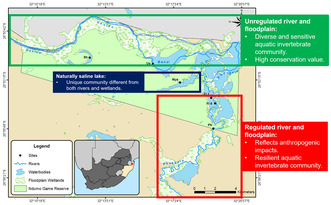

## 1 INTRODUCTION

Natural flooding is a key driver of aquatic community composition as it stimulates spawning and migratory behaviour in many fish species and creates nursery and refuge areas for aquatic biota in the created floodplain reaches (Greenwood & Booker, 2015; Macdonald & Crook, 2014; Naus & Reid Adams, 2018; Rocha et al., 2012). Globally, the majority of rivers are regulated through large impoundments for anthropogenic reasons such as water storage, abstraction and supply for hydropower plants (Grill et al., 2019; Jones et al., 2021; Nilsson et al., 2005). This alters the physical structure and function of a river by modifying flood regimes, which affects the biological and chemical composition of the ecosystem (Nilsson et al., 2005; Poff & Zimmerman, 2010; Schneider & Petrin, 2017; Watson & Dallas, 2013). River regulation and impoundments affect water quality through sediment and nutrient retention (de Necker, Neswiswi, et al., 2020; Grill et al., 2019; van Rooyen et al., 2022). Changes in water quality, in conjunction with modified flow, may lead to shifts in the composition of native aquatic communities, including fish and invertebrates both upstream and downstream of the dam, while also increasing the success of invasive species including aquatic molluscs, fish and macrophytes (Beatty et al., 2017; Brooks et al., 2011; Chanut et al., 2020; Dube et al., 2019; Milner et al., 2019; Schneider & Petrin, 2017; White et al., 2017).

Traits (e.g., feeding behaviour, respiration, mobility and dispersal mechanism), characterise how organisms interact with their environment (Odume, 2020; Townsend & Hildrew, 1994). These traits therefore determine an organism's ability to resist or adapt to environmental change, including anthropogenic alterations to the physical and/or chemical environment (de la Fuente et al., 2018; Li et al., 2019; Townsend & Hildrew, 1994; van Looy et al., 2018). As a result, species will only survive in a habitat if they possess the appropriate combinations of traits (Akamagwuna et al., 2022; Mondy & Usseglio‐Polatera, 2014; Odume et al., 2023). Traits can therefore be used in both short‐ and long‐term biomonitoring and ecological research to assess various anthropogenic effects (de la Fuente et al., 2018; Mondy et al., 2016; Odume, 2020; Townsend & Hildrew, 1994).

Aquatic invertebrates are an essential component of aquatic ecosystems, influencing ecosystem processes in various ways including periphyton control and acting as a food source for organisms at higher trophic levels (Chanut et al., 2020; de Necker et al., 2016; Majdi et al., 2022; Majdi & Traunspurger, 2017). These organisms encompass a diverse group of species with a widespread distribution and range of traits and adaptations to ensure survival in various aquatic environments (de Necker et al., 2021; de Necker, Brendonck, et al., 2022; Dube et al., 2022; Jeffries et al., 2016; Lake, 2011; Wantzen et al., 2016). Furthermore, aquatic invertebrates are known to undergo shifts in community composition in response to anthropogenic stressors (Akamagwuna et al., 2022; Cai et al., 2017; Foster et al., 2015; Helson & Williams, 2013; Magbanua et al., 2010; Mangadze et al., 2019; Ntloko et al., 2021; Villastrigo & García‐Criado, 2017). As a result, aquatic invertebrate traits and sensitivities have successfully been used as a biomonitoring method to assess ecosystem health (Akamagwuna et al., 2022; de Necker et al., 2023; Magbanua et al., 2010; Mangadze et al., 2019; Ntloko et al., 2021; Odume, 2020). It is therefore crucial to consider aquatic invertebrate traits and ecological preferences in freshwater studies to help understand community structure and disturbance effects and ensure maintenance of ecological processes (Akamagwuna et al., 2022; Vinagre et al., 2017).

Anthropogenic and climate‐driven alterations to these natural flooding/drying cycles pose a significant risk to the community composition of aquatic invertebrates, which may result in not only a loss or extinction of species, but also in alterations in the food web structure of these ecosystems (Batzer & Boix, 2016; Covich et al., 1999; de Necker, Brendonck, et al., 2022; Dube et al., 2022; Dudgeon et al., 2006; Jeffries et al., 2016; Zimmer et al., 2016). It is therefore essential to monitor the community composition of all aquatic biota in regulated ecosystems that experience climate‐driven drought events.

Several studies conducted worldwide, including Sweden (Zhang et al., 1998), the USA (Merritt & Cooper, 2000; Steel et al., 2018), Portugal (Cortes et al., 2002), Canada (Macnaughton et al., 2017) and Chile (Banegas‐Medina et al., 2021), have consistently demonstrated a distinction between flow‐regulated through the construction of impoundments, and rivers that are not regulated in such a manner. All of these studies have indicated a negative effect of river regulation on factors such as species abundance, diversity and the presence of sensitive taxa. Furthermore, studies have also found alterations and declines in aquatic invertebrate diversity, community composition and functional traits within regulated and anthropogenically influenced streams and riverine systems (Gallardo et al., 2008, 2014; Kuiper et al., 2014). However, there is a scarcity of research in Africa, despite being one of the most affected continents in terms of anthropogenic use of freshwater systems (Akamagwuna et al., 2022; Ntloko et al., 2021; Odume, 2020).

**FIGURE 1:**
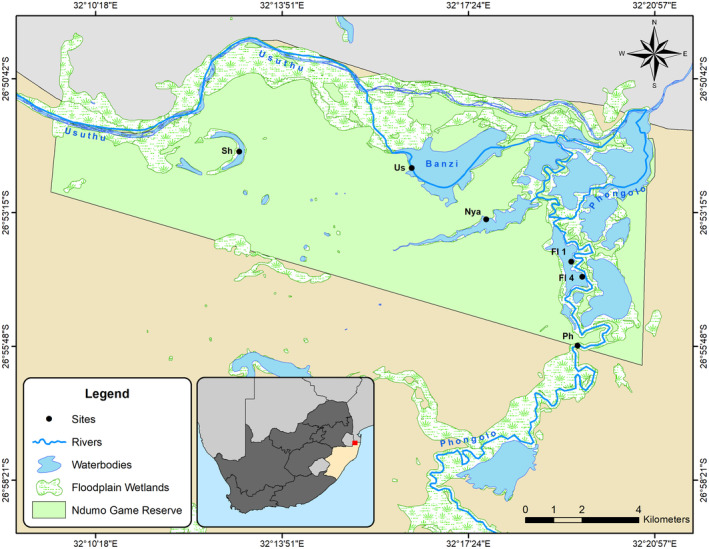
Map of the study area within the Ndumo Game Reserve where aquatic invertebrates were collected during the present study (Usuthu River, Lakes Shokwe and Nyamithi) together with sampling sites from previous surveys (Phongolo River, Phongolo floodplain wetlands 1 and 4 and Lake Nyamithi).

The lower Phongolo River and floodplain (PRF), located in northern KwaZulu‐Natal, is one of South Africa's largest and most important floodplain ecosystems (de Necker et al., 2021; de Necker, Brendonck, et al., 2022; de Necker, Manfrin, et al., 2020; de Necker, Neswiswi, et al., 2020; Dube et al., 2015, 2019). This river and floodplain serve important irrigation, domestic and livestock watering purposes for local communities, while also supporting some of the highest aquatic biodiversity in South Africa (Acosta et al., 2020; Smit et al., 2016). The PRF is fully dependent on controlled flood releases for fresh water, receiving annual controlled flooding regimes from the Pongolapoort Dam, located upstream of the floodplain (de Necker, Neswiswi, et al., 2020; Dube et al., 2015) (Figure S1). On the contrary, the Usuthu River and its associated floodplain is a largely unregulated river, with only small weirs located in the upper catchment of Eswatini, but no large impoundments that regulate its natural flow (Kowalkowski et al., 2007; Vilane & Tembe, 2016; Figure S1). Although the Usuthu River is also used for irrigation, domestic purposes, livestock watering, industrial activities, tourism and fish farming (Kowalkowski et al., 2007; Vilane & Tembe, 2016), the human‐driven activities are present to a much lesser degree than that present in the PRF (Hendriks & Rossouw, 2009; van Rooyen et al., 2022; Figure S1). These two rivers are important water sources for the floodplain ecosystems of the PRF, including the two largest floodplain lakes (Lake Nyamithi and Lake Shokwe) of the Ndumo Game Reserve (NGR), the only conservation area of the PRF (Figure 1). These two rivers merge and form a confluence within the NGR. These floodplain habitats are important for the breeding, spawning and foraging of many aquatic biota (Acosta et al., 2020; de Necker et al., 2021; de Necker, Gerber, et al., 2022; van Rooyen et al., 2022). As such, both the Phongolo and Usuthu rivers are important to the continued maintenance of biotic diversity and ecological functioning of these floodplain lakes.

Since fewer disturbances are present on the Usuthu River compared with the Phongolo River (Hendriks & Rossouw, 2009; van Rooyen et al., 2022), the Usuthu River should act as a refuge for aquatic biota of the floodplain systems. Furthermore, the water quality of the Usuthu River is reportedly in a better condition than that of the Phongolo River (de Necker, Neswiswi, et al., 2020; van Rooyen et al., 2022). Consequently, the Usuthu River is expected to support a higher biodiversity of aquatic invertebrates. Whether this is indeed the case, and how the biodiversity between the two systems compares to one another is unclear, as prior research in the PRF has focused largely on the Phongolo River (de Necker, Neswiswi, et al., 2020; Dube et al., 2015; Smit et al., 2016), its associated floodplain and the temporary wetlands of the area (de Necker et al., 2021; de Necker, Brendonck, et al., 2022; de Necker, Gerber, et al., 2022; de Necker, Manfrin, et al., 2020; Dube et al., 2017, 2019, 2020). In contrast, information about aquatic biodiversity and community dynamics of the Usuthu River and its associated floodplain lake (Lake Shokwe) is scarce, with no information on how this compares to that of the Phongolo River and its floodplain wetlands, nor the role of these two rivers in the ecological functioning of Lake Nyamithi. Furthermore, due to drought in South Africa from 2016 to 2018, no flood releases occurred from the Pongolapoort Dam and thus Lake Nyamithi received little to no inflow from the Phongolo River (de Necker et al., 2021; de Necker, Brendonck, et al., 2022; de Necker, Gerber, et al., 2022; de Necker, Manfrin, et al., 2020). Therefore, the lake was primarily dependent on freshwater input and inflow of aquatic biota from the unregulated Usuthu River, particularly in 2018.

The first aim of this study was to determine and compare the diversity of aquatic invertebrate communities between an impacted and regulated river to a less impacted, unregulated river as well as their associated floodplains to establish whether the aquatic invertebrate community reflects that of a less impacted system that is in a better condition than that of the impacted river. The second aim of this study was to determine whether the invertebrate communities of a lake that receives water from both the regulated and unregulated river would exhibit a stronger similarity of its invertebrate communities to either of the rivers.

The hypotheses for this study were as follows:

1. While the Phongolo River experiences more regulatory influences, these impacts will not result in demonstrable differences in the aquatic invertebrate communities within this river and its associated floodplain wetlands compared to the less impacted and unregulated Usuthu River and its associated floodplain lake.
2. The aquatic invertebrate communities in Lake Nyamithi are expected to exhibit a higher degree of similarity to those found in the Usuthu River, as this river was the primary source of water and aquatic biota in Lake Nyamithi for an extended period during a supraseasonal drought.

To test these hypotheses, the study collected aquatic invertebrate communities and water quality data from a regulated river (Phongolo River), and an unregulated river (Usuthu River), as well as their respective floodplain wetland and lake systems.

## 2 MATERIALS AND METHODS

### 2.1 Site description

The lower Phongolo River and its associated floodplain (PRF) starts below the Pongolapoort Dam at the town of Jozini (de Necker, Neswiswi, et al., 2020; Merron et al., 1993; Smit et al., 2016). The Pongolapoort Dam was constructed in 1973 with the primary purpose of providing water for commercial cotton and sugarcane crops (de Necker, Neswiswi, et al., 2020; Hendriks & Rossouw, 2009). Downstream of the dam, the lower Phongolo River (hereafter Phongolo River (PR)) extends approximately 80 km downstream, covering a catchment of approximately 120 km^2^, while the floodplain covers up to 13,000 ha when fully inundated. This river flows through NGR to the confluence of the Usuthu River, before entering Mozambique as the Rio Maputo River (de Necker, Neswiswi, et al., 2020; Dube et al., 2015; Jaganyi et al., 2009; NGR IMP, 2009). The water quality and biodiversity of the PRF have been impacted by anthropogenic practices including industry, forestry, agriculture and mining activities for several decades, as well as flood mismanagement from the Pongolapoort Dam (de Necker, Neswiswi, et al., 2020). Upstream of the dam, the Phongolo River has been utilised for various commercial agricultural purposes. Below the dam, the PRF serves as a source of water for both commercial and subsistence agriculture as well as household use for rural communities (Figure S1). Prior to dam construction, the PRF was subjected to natural summer flooding that created a mosaic of environments for aquatic biota (de Necker, Neswiswi, et al., 2020; Dube et al., 2015). At present, the flooding regimes of the PRF lack ecological foundation and are determined by consultations with local stakeholders to determine the needs of the agricultural sector and local communities as well as the volume of water present in the Pongolapoort Dam (de Necker, Neswiswi, et al., 2020; Dube et al., 2015). To date, the flood release largely consists of a small flood release in June and a larger flood release occurring in October, although annual variations may occur (see Figure S2 for a detailed hydrograph).

The Phongolo and Usuthu rivers both flow into, and form part of, Ndumo Game Reserve (NGR) the only conserved area of the PRF (de Necker, Neswiswi, et al., 2020; van Rooyen et al., 2022; Figure 1). The Usuthu River (Us) originates in Mpumalanga before flowing through Eswatini into KwaZulu‐Natal. It is a largely unregulated river with a catchment area of approximately 2682 km^2^ covering most of the Eswatini region (Vilane & Tembe, 2016). This is the largest river that flows through Eswatini and adds significant value to the Eswatini economy (Mthimkhulu, 2018). Various commercial activities are located along the Usuthu River, impacting the river through changes in water quality (Magagula et al., 2010). However, these are to a far lesser degree compared to alterations observed in the Phongolo River (van Rooyen et al., 2022; Figure S1). Since the Usuthu River is not regulated, its flooding regime is also typical of free‐flowing rivers, largely natural and controlled by climatic conditions. Within NGR, forms the northern boundary of the reserve and the northern border with Mozambique (NGR IMP, 2013; van Rooyen et al., 2022; Whittington et al., 2013; Figure 1). This river is the primary source of water to Lake Shokwe and also supplies water to Lake Nyamithi (Birkhead et al., 2018; de Necker et al., 2021).

Lake Shokwe (Sh) is an oxbow lake with a closed drainage system and is located within the NGR (Figure 1). This lake is the first of a series of floodplain lakes that receive water from the Usuthu River through flooding after rainfall within the river catchment (van Rooyen et al., 2022). Lake Nyamithi (Nya) is a naturally saline floodplain lake (salinity ±3 g/L) located in the NGR (Figure 1; de Necker et al., 2021) and is the largest semi‐permanent floodplain lake within the reserve (183.4 ha; Calverley & Downs, 2014; Heeg & Breen, 1982). This lake is of high ecological importance, as 59 species of wetland‐dependent birds have been recorded in and around the lake, while it is also home to a large population of hippopotami and the third largest population of crocodiles in South Africa (Calverley & Downs, 2014; de Necker et al., 2021). The lake receives much of its water during the high flow periods through flooding of the Phongolo River following flood releases from the Pongolapoort Dam, while localised rainfall in the catchment and back flooding from the Usuthu River also contribute to its water supply (de Necker, Brendonck, et al., 2022; van Rooyen et al., 2022).

Two floodplain wetlands (FL 1 and FL 4; Figure 1) situated within the NGR in close proximity to each other and the Phongolo River (within 2 km) were selected for comparison with the aquatic invertebrate communities of Lake Shokwe. These wetlands were selected as they represent typical tropical floodplain wetlands that receive water through rainfall and connection with adjacent rivers during flood releases in the wet season, and typically dry out during the dry season when they become disconnected from the rivers (Wantzen et al., 2016). These two wetlands comprised shallow water levels, muddy substrates, and abundant floating, emergent and marginal vegetation, as described in previous studies (see de Necker, 2019; de Necker, Gerber, et al., 2022; Dube et al., 2017). These are identical attributes associated with Lake Shokwe.

During the current study, aquatic invertebrates and water for chemical analyses were collected in 2018 twice from Lake Shokwe, once during high flow (June) and once during low flow (November) and once from the Usuthu River and Lake Nyamithi during low flow. Samples were collected from different areas and habitats of each site to ensure a comprehensive representation of each site. The collected data (2018) were compared with those collected during previous studies from the Phongolo River inside the NGR (high flow 2012, high flow 2013: Smit et al., 2016; low flow 2016: de Necker, 2019), two Phongolo River floodplain wetlands within the NGR (high flow 2014: Dube et al., 2019) and on Lake Nyamithi (low flow 2017: de Necker et al., 2021; refer to Table 1). Each of the sites from these previous surveys were sampled once during each survey and all the sites sampled during the current study in 2018 were identical to those sampled in the previous surveys (Figure 1).

**TABLE 1:** Different locations sampled during a range of surveys inside and outside the Ndumo Game Reserve on the Phongolo and Usuthu river systems.

Sampling site	Study collected	Survey year (flow)	Site code
Usuthu River	Current study	November 2018 (High flow)	Us 18
Phongolo River	de Necker (2019)	September 2016 (Low flow)	Ph 16
Lake Shokwe	Current study	June 2018 (High flow)	Sh HF
		November 2018 (Low flow)	Sh LF
Lake Nyamithi	de Necker et al. (2021)	November 2017 (Low flow)	Nya 17
Lake Nyamithi	Current study	November 2018 (Low flow)	Nya 18
Phongolo Floodplain Lake 1	Dube et al. (2019)	February 2014 (High flow)	FL 1
Phongolo Floodplain Lake 4	Dube et al. (2019)	February 2014 (High flow)	FL 4
Phongolo River	Smit et al. (2016)	November 2012 – April 2013 (High flow)	n/a

### 2.2 Field sample collection

Temperature (°C), pH, electrical conductivity (EC: μS/cm), total dissolved solids (TDS: mg/L) and oxygen concentration (DO: mg/L) were measured in situ at each site using Extech EC600 pH/conductivity and Extech D0600 dissolved oxygen multiparameter meters. Integrated subsurface water samples were collected at each site and frozen until further analysis for selected parameters.

Aquatic invertebrates were collected from all available biotopes (vegetation, stones in and out of current where necessary and gravel, sand and mud) at each of the sites using a standard 30 cm × 30 cm square metal frame net with a 500‐μm mesh. A standard of 40 net sweeps were taken at each sampling site after which the integrated invertebrate sample was placed in a 500‐mL polyethylene jar and fixed with 70% ethanol.

### 2.3 Laboratory analysis

In the laboratory, water samples were left to thaw and reach room temperature and subsequently analysed using Merck photometric test kits and a Merck Pharo 100 Spectroquant (method as in van Rooyen et al., 2022). Samples were analysed for the following variables (corresponding test kit protocol numbers in parentheses): nitrate (NO_3_‐N, 109713), nitrite (NO_2_‐N, 114776), sulphate (${\mathrm{SO}}_{4}^{2-}$, 114791), turbidity (measured in NTU), chemical oxygen demand (COD, 101796), chloride (Cl, 114897), ammonium (NH_4_‐N, 114752), orthophosphate (${\mathrm{PO}}_{4}^{3-}$‐P, 114848) and total hardness (TH, 100961).

### 2.4 Aquatic invertebrate identification

In the laboratory, aquatic invertebrates were washed under running tap water in a 250‐μm mesh size sieve to remove the ethanol along with any mud and large pieces of debris. All aquatic invertebrates were identified to the lowest possible taxonomic level with appropriate identification guides (Day et al., 1999, 2003; Day & de Moor, 2002a, 2002b; Day, De Moor, et al., 2001; Day, Stewart, et al., 2001; de Moor et al., 2003a, 2003b; Stals & de Moor, 2008) using a Nikon Model C‐LEDS dissection microscope and counted.

### 2.5 Aquatic invertebrate traits

Following aquatic invertebrate identification, taxa were classified according to their different traits (functional feeding groups, habitat preference, dispersal mode, mode of respiration, aquatic life stage and hydraulic preference) by using the South African Macroinvertebrate Trait Database and appropriate guides (Fry, 2021; Odume et al., 2018, 2023) (see Appendix Table S1). Aquatic invertebrate families were further classified according to their sensitivity or tolerance to environmental change (change in flow and habitat), water quality and/or pollution. Sensitivity scores were obtained from scores determined by Dickens and Graham (2002). The sensitivity scores were separated into two main categories namely tolerant (sensitivity score 0–7) and sensitive (score 8–15) families.

### 2.6 Data analysis

Aquatic invertebrate and water quality data from Dube et al. (2019) (FL 1 and FL 4) and de Necker et al. (2021) (Nya 17) and the unpublished chapters from the Ph.D. thesis by de Necker (2019) (Ph 16) were used together with the data from the current study to compare the aquatic invertebrate communities of different aquatic ecosystems of the NGR to achieve aims 1 and 2 of this study. To ensure comparability, the same sampling techniques and analyses as those in the previous studies were used.

All data analyses regarding the aquatic invertebrates were performed on the lowest possible level of taxonomic identification. Untransformed data were used to ascertain the number of taxa, number of individuals and to calculate Pielou's Evenness index (evenness) and Shannon‐Wiener diversity (diversity) for each site. Aquatic invertebrate count data were normalised by means of square root (√) transformation to reduce the effect of dominant taxa while water quality data, excluding pH, were log‐transformed [*y* = log (*x* + 1)] prior to the constrained analysis (de Necker et al., 2021).

A principal component analysis (PCA) of all aquatic invertebrate taxa from all sampling sites was created using Canoco v5 This PCA was used to determine whether any of the aquatic invertebrate taxa were associated with a particular site or survey. A second PCA was created to determine whether any of the traits determined for the aquatic invertebrates collected were associated with a particular site or survey. Additionally, a constrained canonical correspondence analysis (CCA) was used to compare the Usuthu River and its associated floodplain lake (Lake Shokwe) with the Phongolo River and its associated floodplain wetlands (FL 1 and 4). These two PCA's and the aforementioned CCA were done to achieve aim 1. A second CCA was performed since Lake Nyamithi receives water input from both the Usuthu and Phongolo rivers and was used to compare the aquatic invertebrate communities of the Usuthu River, Phongolo River and Lake Nyamithi to determine whether the aquatic invertebrate communities of this lake are more associated with either of these two riverine ecosystems, as a means to achieve Aim 2 of this study. Prior to CCA analyses, water variables were tested for possible collinearity between variables, and in the case of highly collinear variables (>10), they were excluded from the analysis (methods as in de Necker et al., 2021; Zuur et al., 2009). Due to high multicollinearity, only TH, NO_3_, ${\mathrm{PO}}_{4}^{3-}$ and ${\mathrm{SO}}_{4}^{2-}$ were included for the CCAs.

The percentage (%) that aquatic invertebrates contributed to dissimilarities between the Usuthu and Phongolo rivers was calculated with a similarity percentage analysis (SIMPER) using the Bray–Curtis dissimilarity matrix in Primer v7. This analysis was further used to calculate the contribution of aquatic invertebrates to dissimilarities between Lake Shokwe (the floodplain lake associated with the Usuthu River) and the two floodplain wetlands associated with the Phongolo River (FL 1 and FL 4). These SIMPER analyses were used to achieve Aim 1 of this study.

To determine the sensitive and tolerant taxa occurring in the different systems, pie charts were created to compare aquatic invertebrate family sensitivity data from the Phongolo River collected during surveys in 2012–2013 (Smit et al., 2016) and 2016–2017 (de Necker, 2019) to the Usuthu River (2018; present study).

## 3 RESULTS

### 3.1 Aquatic invertebrate community structures

A total of 131 taxa (68 families) were collected from all sampling sites during the present study as well as the previous surveys. 33 taxa (30 families) were identified from the Usuthu River, while the Phongolo River had a total of 38 taxa (29 families) (Table S2). The most abundant taxa in the Usuthu River were Baetidae, *Rhagovelia* sp. (Family Veliidae), *Caridina nilotica* (Family Atyidae) and *Aulonogyrus* sp. (Gyrinidae). The most abundant taxa in the Phongolo River were Chironomidae, *Thermocyclops* sp. (Cyclopidae), Chydoridae, and this was the only site with the native mollusc *Radix natalensis* (Family Lymnaeidae) and the invasive snail *Physella acuta* (Family Physidae).

A total of 23 taxa (17 families) were collected in Lake Shokwe during the LF survey, while 48 taxa (30 families) were collected in the HF survey (Table S2). The most abundant taxon in Lake Shokwe during both the LF survey and the HF survey was *Micronecta* sp. (Family Corixidae). A total of 32 different taxa (18 families) were found in FL 1 and 29 taxa (18 families) in FL 4 (Table S2). The most abundant taxa in FL 1 were Tanypodinae, *Anisops* sp., Orthocladiinae, Oligochaeta, *Appasus* sp. (Family Belostomatidae), *Cloeon* and *Procloeon* and (Family Baetidae). while in FL 4 the most abundant were *Anisops* sp., Tanypodinae, *Cloeon* and *Procloeon* sp., *Neohydrocoptus* (Family Noteridae), *Appasus* sp. and *Bulinus forskalii* (Family Bulinidae).

In 2017, 30 taxa (19 families) were recorded in Lake Nyamithi, while 34 taxa (26 families) were sampled in 2018 (Table S2). The most abundant taxa in 2017 were Ostracoda, *C. nilotica*, *Hyphydrus* sp. (Family Dytiscidae), *Berosus* sp., *Brachythemis leucosticta* (Family Libellulidae) and *Anisops* sp., and in 2018 were *Micronecta* sp., Ostracoda, Tanypodinae, *Bezzia* sp. (Ceratopogonidae), *Anisops* sp. and Oligochaeta.

**FIGURE 2:**
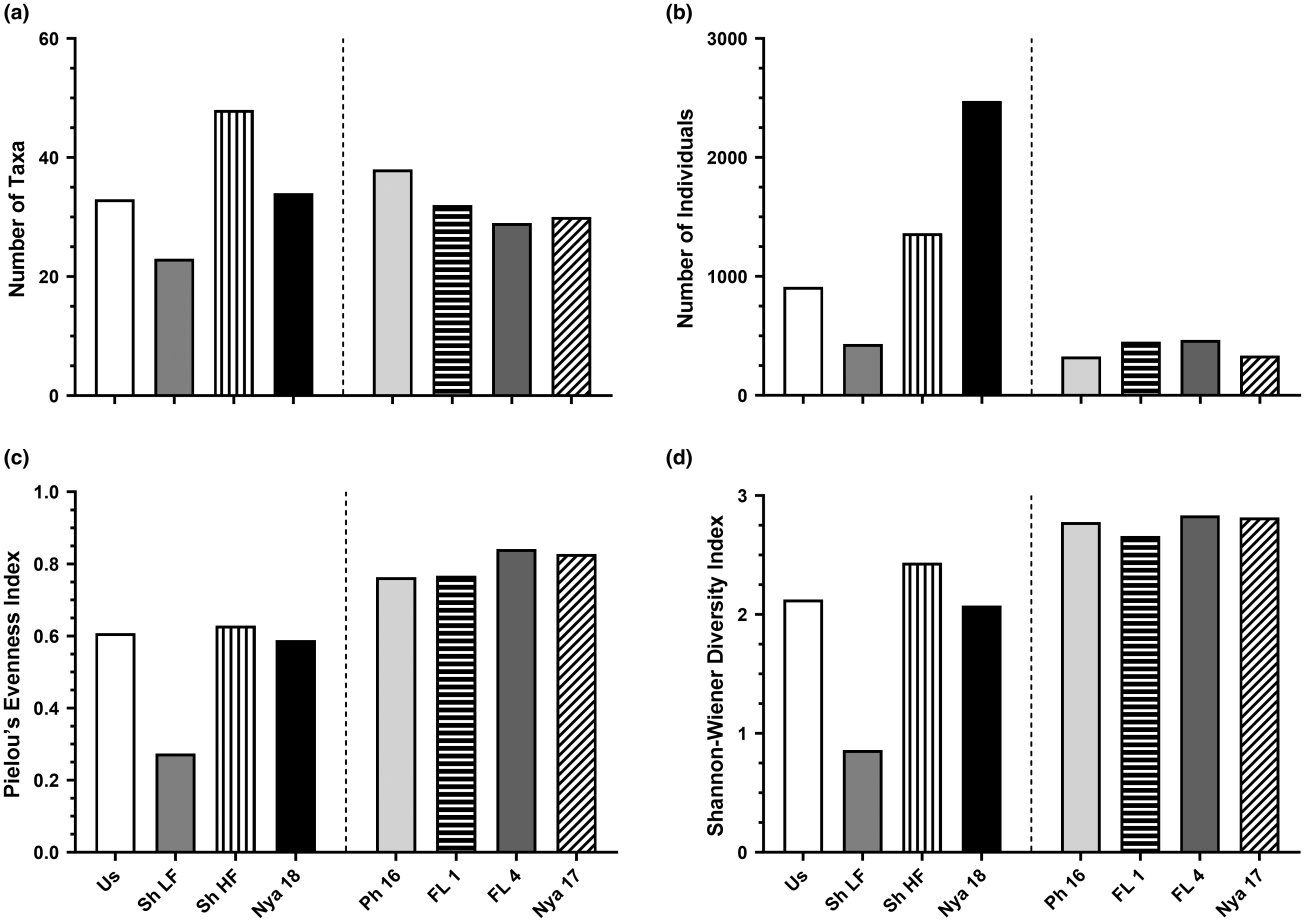
Comparison between (a) number of taxa, (b) number of individuals, (c) Pielou's Evenness Index and (d) Shannon–Wiener Diversity Index collected in the Usuthu River (Us 18), its associated floodplain wetland (Lake Shokwe) in the low flow (Sh LF) and high flow (Sh HF) and Lake Nyamithi (Nya 18) during the current study and the Lower Phongolo River (Ph 16), its associated floodplain wetlands (FL 1 and FL 4) and Lake Nyamithi (Nya 17) sampled during previous studies. Data from the Lower Phongolo River were collected by de Necker (2019) and its associated floodplain wetlands by Dube et al. (2019). The vertical dashed line indicates separation between the sites sampled during the current study and those sampled in previous studies.

The HF survey of Lake Shokwe yielded the highest number of taxa (52), while the greatest number of individuals was collected from Lake Nyamithi in 2018 (2475; Figure 2). The overall evenness and diversity was highest in Phongolo River Floodplain wetland, FL 4 (0.84 and 2.83, respectively). Of all taxa collected at all sites, 16 (12.2%) were unique to the Phongolo River, which was the highest of all sites, while 14 (10.7%) were unique to Lake Shokwe during HF, 11 (8.4%) were unique to the Usuthu River, 7 (5.3%) were unique to Lake Nyamithi sampled in 2017, 6 (4.6%) were unique to Lake Shokwe during LF, 5 (3.8%) each were unique to Lake Nyamithi sampled in 2018 and the other Phongolo River Floodplain wetland (FL 1) while only 3 (2.29%) were unique to FL 4 (Table S3).

### 3.2 Aquatic invertebrate community comparison between sampled floodplain systems

The SIMPER analysis comparing the Usuthu and Phongolo rivers indicated an 89.85% dissimilarity in the structure of the macroinvertebrate community between the two rivers (Table 2). This was as a result of 46 taxa of which 31 contributed ≥1% to the differences between these two sites. The taxa that contributed the most to these differences (more than 20 individuals) were *Aulonogyrus* sp., Baetidae and *C. nilotica*, which were highest in the Usuthu River, as well as several that were present only in the Usuthu River and only in the Phongolo River (see Table 2).

**TABLE 2:** Percentage contribution of the 31 aquatic macroinvertebrate taxa that contributed ≥1% to dissimilarities between the Usuthu River and the Phongolo River calculated by Similarity of percentage analysis (SIMPER).

Site	Overall dissimilarity (%)	Species	Average dissimilarity (%)	Percentage contribution (%)	Cumulative contribution (%)
Usuthu vs Phongolo	89.85%	Baetidae	7.79	8.67	8.67
		*Rhagovelia* sp.	6.33	7.05	15.72
		*Aulonogyrus* sp.	4.66	5.19	20.9
		Chironominae	4.49	5	25.9
		*Caenis* sp.	4.13	4.59	30.5
		*Caridina nilotica*	3.56	3.96	34.45
		*Thermocyclops* sp.	3.38	3.76	38.22
		*Afrocaenis* sp.	2.75	3.06	41.27
		Chydoridae	2.56	2.85	44.13
		*Afronurus* sp.	2.42	2.69	46.81
		*Appasus* sp.	2.21	2.45	49.27
		*Pseudagrion* sp.	2.03	2.26	51.53
		*Tetrathemis polleni*	2.03	2.26	53.8
		*Culex* sp.	1.71	1.9	55.7
		*Physella acuta*	1.64	1.82	57.52
		*Simocephalus serrulatus*	1.64	1.82	59.34
		*Corbicula fluminalis*	1.56	1.74	61.07
		*Nilus margartatus*	1.56	1.74	62.81
		*Laccocoris* sp.	1.39	1.55	64.36
		*Allocnemis leucosticta*	1.3	1.45	65.81
		Cyprididae	1.3	1.45	67.27
		Darwinulidae	1.3	1.45	68.72
		*Hydropsyche* sp.	1.3	1.45	70.17
		*Laccobius* sp.	1.3	1.45	71.62
		*Cloeon & Procloeon*	1.1	1.23	72.85
		Oligochaeta	1.1	1.23	74.08
		*Anax* sp.	0.99	1.1	75.17
		*Berosus* sp.	0.99	1.1	76.27
		*Cybister* sp.	0.99	1.1	77.37
		*Enochrus* sp.	0.99	1.1	78.47
		*Paragomphus* sp.	0.99	1.1	79.57

The SIMPER analysis indicated a large dissimilarity (80.26%) between Lake Shokwe (LF and HF) and the two floodplain wetlands associated with the Phongolo River (FL 1 and FL 4; Table 3). This was as a result of 53 different aquatic invertebrate taxa of which 34 contributed ≥1% to the differences between the two sites. These included *Agraptocorixa* sp., Baetidae, *Enithares* sp., Oligochaeta, *Mesovelia* sp. and *Enochrus* sp., which were most abundant in Lake Shokwe, and Tanypodinae, *Anisops* sp., *Hydrophilus* sp., *C. nilotica*, *Neogerris* sp., *Anax* sp. and Chironominae that were highest in the two floodplain wetlands (FL 1 and FL 4). Additionally, several taxa were exclusively found in either Lake Shokwe or FL 1 and 4 (see Table 3).

**TABLE 3:** Percentage dissimilarities contribution of the 34 aquatic macroinvertebrate taxa that contributed ≥1% to differences between the Usuthu River‐associated floodplain lake (Lake Shokwe) and the two Phongolo River‐associated floodplain wetlands (FL 1 and FL 4) calculated by Similarity of percentage analysis (SIMPER).

Site	Overall dissimilarity (%)	Species	Average dissimilarities (%)	Percentage contribution (%)	Cumulative contribution (%)
Shokwe vs Floodplain wetlands (FL 1 and FL 4)	80.26%	*Micronecta* sp.	10.28	12.81	12.81
		*Agraptocorixa* sp.	3.05	3.8	16.61
		Tanypodinae	3	3.74	20.35
		*Cloeon* and *Procloeon*	2.68	3.34	23.69
		*Anisops* sp.	2.59	3.23	26.92
		*Appasus* sp.	2.46	3.07	29.99
		Baetidae	2.25	2.81	32.8
		Orthocladiinae	2.13	2.66	35.46
		*Bulinus forskalii*	1.83	2.28	37.74
		*Enithares* sp.	1.8	2.24	39.98
		Oligochaeta	1.8	2.24	42.22
		*Bulinus tropicus*	1.78	2.21	44.43
		*Neohydrocoptus* sp.	1.78	2.21	46.65
		*Laccophilus* sp.	1.74	2.17	48.81
		*Lethocerus niloticus*	1.68	2.1	50.91
		*Hyphydrus* sp.	1.65	2.05	52.96
		*Pirata* sp.	1.51	1.88	54.84
		Hydrachnellae	1.34	1.67	56.51
		*Hydrophilus* sp.	1.32	1.64	58.15
		*Caridina nilotica*	1.25	1.56	59.71
		*Neogerris* sp.	1.19	1.48	61.2
		*Canthydrus* sp.	1.19	1.48	62.68
		*Mesovelia* sp.	1.17	1.45	64.13
		*Enochrus* sp.	1.1	1.37	65.5
		*Enallagma* sp.	1.04	1.3	66.8
		*Anax* sp.	0.95	1.19	67.99
		*Ranatra* sp.	0.93	1.16	69.15
		*Parasthetops* sp.	0.91	1.14	70.29
		*Neumania* sp.	0.9	1.12	71.41
		*Hydaticus* sp.	0.89	1.11	72.51
		Chironominae	0.88	1.1	73.61
		*Pseudagrion* sp.	0.84	1.05	74.66
		Dytiscidae larvae	0.83	1.03	75.69
		*Regimbartia* sp.	0.8	1	76.69

**FIGURE 3:**
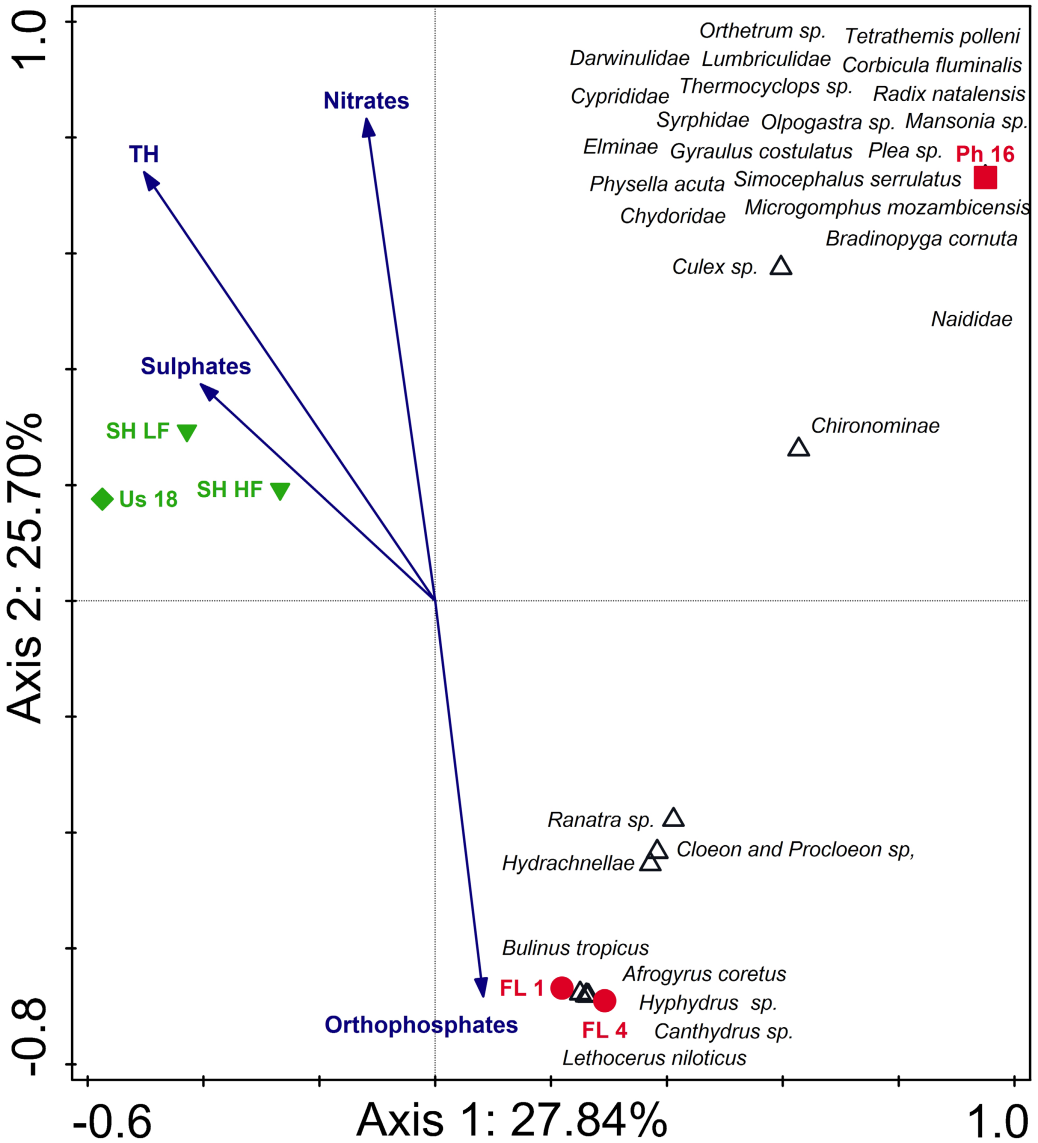
Canonical correspondence analysis triplot of the 30 best‐fitting aquatic macroinvertebrate taxa and selected water nutrients (blue arrows) of both rivers (Usuthu and Phongolo rivers) and their associated floodplain wetlands (Lake Shokwe, FL 1 and FL 4) collected during the current and previous studies. The triplot explains 53.54% of the total variation in the data with axis 1 explaining 27.84% while 25.70% is explained on axis 2. Usuthu River (Us 18) = green diamond; Lake Shokwe (Sh LF and Sh HF) = green down triangle; Phongolo River (Ph 16) = red square; Phongolo floodplain wetlands (FL 1 and FL 4) = red circle; LF = Low flow, HF=High flow. Data from the Lower Phongolo River were collected by de Necker (2019).

The CCA triplot comparing the two river systems with their associated floodplain wetlands indicated differences between the rivers and their lakes, as well as differences between the lakes (Figure 3). The triplot explains 53.54% of the total variation on the first two axes of which separation on axis 1 (explaining 27.84% of the data variation) is largely as a result of the Usuthu River and its associated floodplain lake (Lake Shokwe) separated from all other sites. As indicated in the SIMPER analyses, these systems differed greatly from one another in their aquatic invertebrate community structures (more than 85%) with several taxa associated exclusively with each of the rivers and lakes, respectively. Additionally, the water quality variables ${\mathrm{SO}}_{4}^{2-}$ (sulphates), ${\mathrm{NO}}_{3}^{-}$ (nitrates) and total hardness (TH) were more associated with the Usuthu River and Lake Shokwe as these variables were much higher in this river and its lake than in the other systems (Table S4). The second axis (explaining 25.70% of the data variation) resulted from the Phongolo River‐associated floodplain wetlands (FL 1 and FL 4) that separated from all other sites. This was due to a difference in the aquatic invertebrate community structures as well as a stronger association between these systems and ${\mathrm{PO}}_{4}^{3-}$ (orthophosphates). These two lakes had an overall higher diversity than other sites and several taxa were found exclusively in these two systems.

**FIGURE 4:**
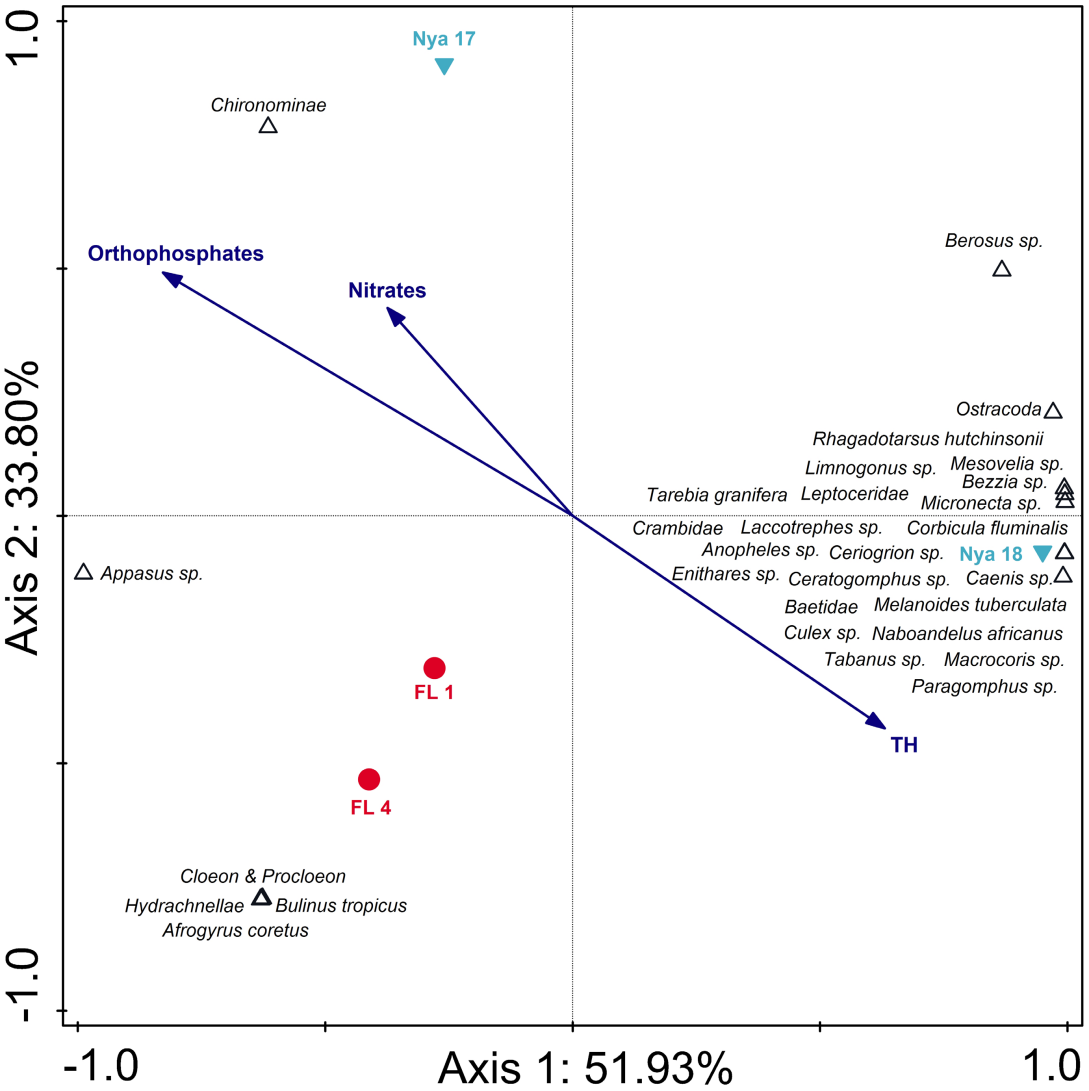
Redundancy analysis triplot of the 30 best‐fitting aquatic macroinvertebrate taxa and selected water nutrients (blue arrows) of Lake Nyamithi (Nya 17 and Nya 18) and floodplain wetlands associated with the Phongolo River (FL 1 and FL 4) collected during the current and previous studies. The triplot explains 85.73% of the total variation in the data with axis 1 explaining 51.93% while 33.80% is explained on axis 2. Phongolo floodplain wetlands (FL 1 and FL 4) = red circle; Lake Nyamithi (Nya 17 and Nya 18) = blue down triangle. Data from the floodplain wetlands and Lake Nyamithi (Nya 17) were collected by de Necker (2019) and de Necker et al. (2021), respectively.

The RDA triplot comparing Lake Nyamithi to the two floodplain wetlands associated with the Phongolo River (FL 1 and FL 4) indicated clear differences between the floodplain wetlands and Lake Nyamithi, as well as temporal variations in Lake Nyamithi (Figure 4). The triplot explained 85.73% of data variation on the first two axes. Variation on axis 1 (explaining 51.93%) was as a result of higher aquatic invertebrate diversity and higher total hardness (TH), in Lake Nyamithi during the present study (Nya 18) compared with the other sites. Separation along axis 2 (explaining 33.80% of data variation) was a result of differences in aquatic invertebrate biodiversity and water quality between the two floodplain wetlands and Lake Nyamithi, particularly at site Nya 17. In 2017, Lake Nyamithi exhibited a similar diversity to the floodplain wetlands, but the composition was markedly different, with limited biota shared between these ecosystems. Orthophosphates and nitrates were negatively associated with FL 1 and FL and were also highest in these two wetlands (Table S4).

### 3.3 Community comparisons between the rivers and Lake Nyamithi

The abundance of sensitive families decreased in the Phongolo River from 2012/2013 (37%; Smit et al., 2016) to 2016 (22%; de Necker, 2019; Figure S2a,b) and the abundance of sensitive families in the Usuthu River (Figure S2c) was similar (36%) to that reported by Smit et al. (2016) (37%). The number of tolerant families in the Phongolo River increased from 63% in 2012/2013 to 78% in 2016 and was much higher than the Usuthu River (64%).

From the rivers and Lake Nyamithi, a total of 94 aquatic invertebrate taxa were collected of which the Usuthu River and Lake Nyamithi shared only 13 taxa (13.83%) and the Phongolo River and Lake Nyamithi shared 15 taxa (15.96%). Taxa only associated with Lake Nyamithi included *Ceratogomphus* sp., *Macrocoris* sp., *Melanoides tuberculata*, *Limnogonus* sp., *Naboandelus africanus*, *Rhagadotarsus hutchinsonii* and *Tarebia granifera*.

The SIMPER comparing the Usuthu River with Lake Nyamithi indicated a distinct difference in aquatic invertebrate communities between the two systems (82.16%; Table S5). A total of 46 taxa contributed to these differences of which 28 contributed ≥1%. The taxa contributing to these differences were Baetidae, *Caenis* sp., *C. niloticus* and *Appasus* sp. that were most abundant in the Usuthu River, *Micronecta* sp., *Anisops* sp., Oligochaeta, *Berosus* sp. and *Enithares* sp., that were highest in Lake Nyamithi as well as several taxa found uniquely in the Usuthu River and in Lake Nyamithi (see Table S5).

The SIMPER comparing the Phongolo River to Lake Nyamithi indicated a large difference (82.49%) between the two sites with 52 taxa contributing to these differences of which 34 made a ≥ 1% contribution (Table S6). The taxa contributing to this difference were Chironominae, *Culex* sp., *Pseudagrion* sp., *C. fluminalis* and *Laccobius* sp. that were the most abundant in the Phongolo River, *Micronecta* sp., *Anisops* sp., Baetidae and *C. nilotica*, which were the most abundant in Lake Nyamithi. as well as several present only in the Phongolo River and only in Lake Nyamithi (see Table S6).

**FIGURE 5:**
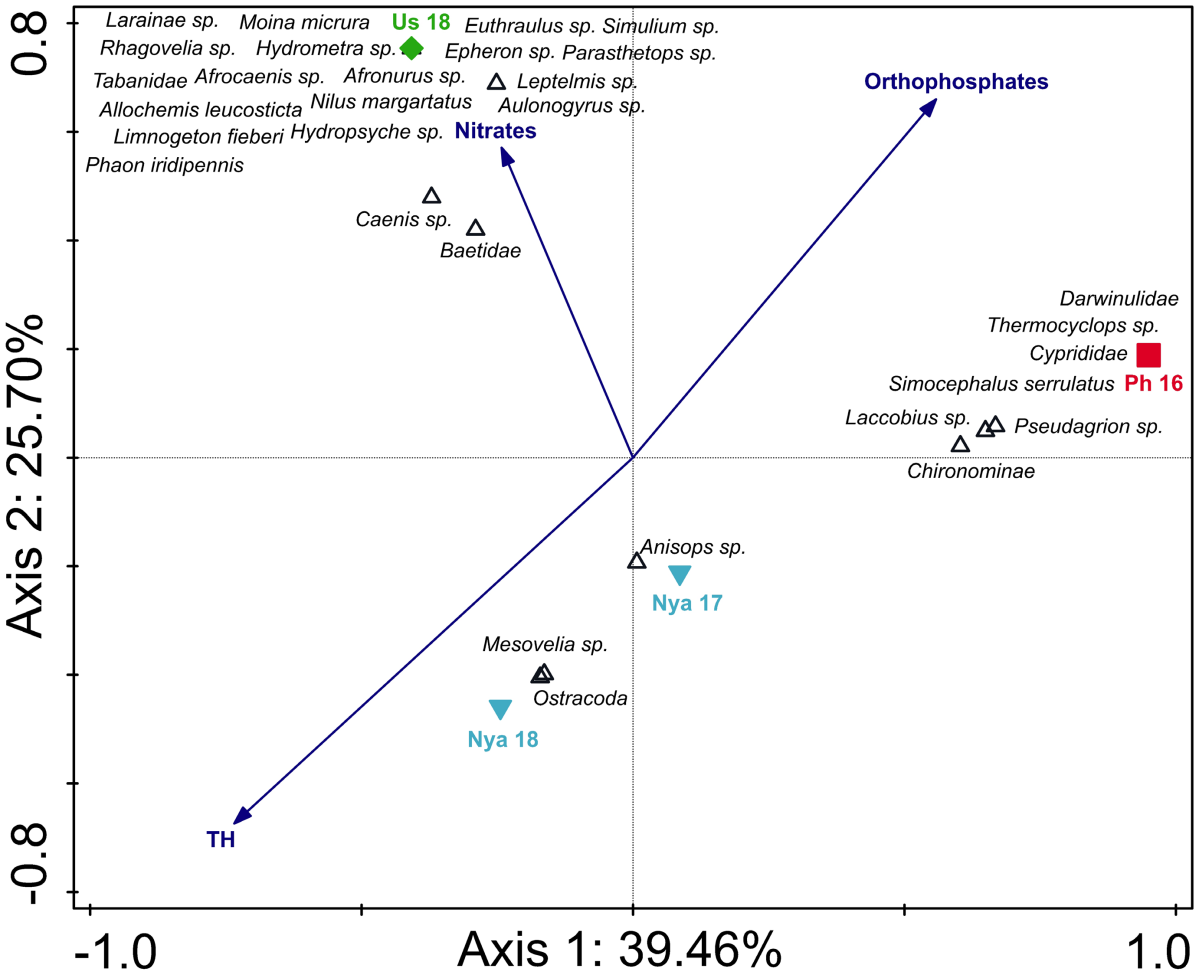
Canonical correspondence analysis triplot of the 30 best‐fitting aquatic macroinvertebrate taxa and selected water nutrients (blue arrows) of both rivers (Usuthu and Phongolo rivers) and Lake Nyamithi (Nya 17 and Nya 18) collected during the current and previous studies. The triplot explains 74.91% of the total variation in the data with axis 1 explaining 39.46% of the variation while 35.45% is explained on axis 2. Usuthu River (Us 18) = green diamond; Lake Shokwe (Sh LF and Sh HF) = green down triangle; Phongolo River (Ph 16) = red square; Lake Nyamithi (Nya 17 and Nya 18) = blue down triangle; LF = Low flow, HF=High flow. Data from the Lower Phongolo River and Lake Nyamithi (Nya 17) were collected by de Necker (2019) and de Necker et al. (2021), respectively.

The CCA triplot comparing the two river systems with Lake Nyamithi explained 74.91% of the data variation on the first two axes (Figure 5). Along axis 1 (explaining 39.46% of data variation) separation occurred due to differences in aquatic invertebrate community structures between the Phongolo River and other sampling sites. The Phongolo River had a high diversity of aquatic invertebrates and several taxa exclusively associated with this site. Axis 2 (explaining 24.48% of data variation) showed separation of the two river sites from Lake Nyamithi as a result of the difference in aquatic invertebrate diversity and abundance between the rivers and Lake Nyamithi as well as key differences in water quality. Lake Nyamithi (both Nya 17 and Nya 18) had the highest levels of total hardness. Conversely, ${\mathrm{PO}}_{4}^{3-}$, which was highest in Nya 18, showed a negative association with the lake, while nitrates, that were highest in the Usuthu River, showed a positive association with this river system.

### 3.4 Aquatic invertebrate trait analysis

**FIGURE 6:**
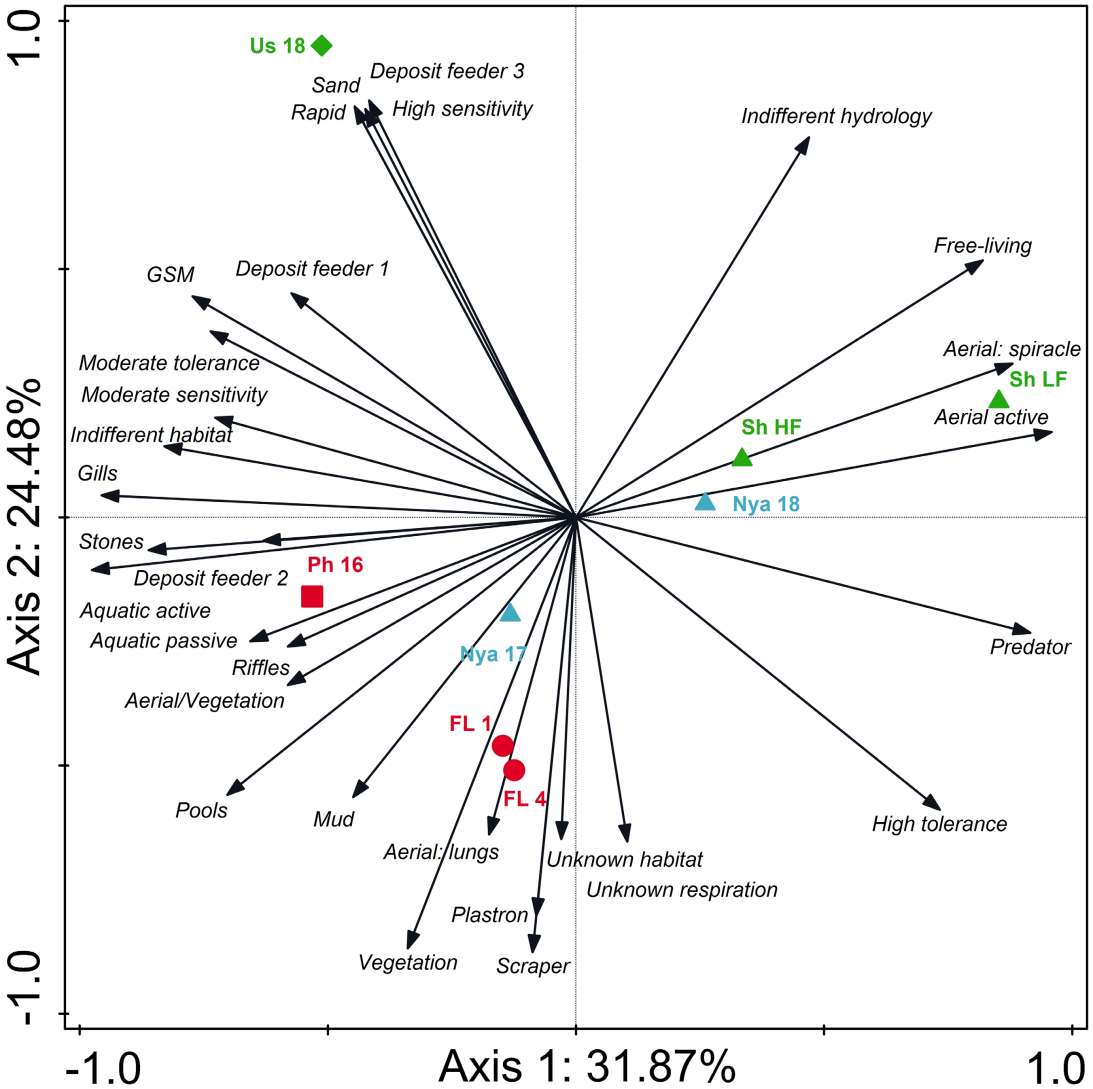
Principal component analysis of the 25 best fitting aquatic invertebrate traits (black arrows) associated with the various sites during the current and previous studies. The first two axes of the biplot explained 56.35% of the total variation in the data with axis 1 explaining 31.87% and axis 2 explaining 24.48%. Usuthu River (Us 18) = green diamond; Lake Shokwe (Sh LF and Sh HL) = green down triangle; Phongolo River (Ph 16) = red square; Lake Nyamithi (Nya 17 and Nya 18) = blue up triangle; Phongolo floodplain wetlands (FL 1 and FL 4) = red circle; LF = Low flow, HF=High flow. Data from the Lower Phongolo River and Lake Nyamithi (Nya 17) were collected by de Necker (2019) and de Necker et al. (2021), respectively.

The PCA comparing aquatic invertebrate traits between the two rivers, associated floodplain wetlands and Lake Nyamithi explained 56.35% of data variation on the first two axes (Figure 6). On axis 1 (explaining 31.87% of data variation) the rivers, particularly the Phongolo River, both floodplain lakes and the two large lakes (Nyamithi and Shokwe) separated from one another based on difference in aquatic invertebrate traits between each of the different systems. Aquatic invertebrate traits linked to the Phongolo River included invertebrates that use both active and passive aquatic dispersal (Aquatic active and Aquatic passive respectively), those that prefer riffle (Riffles) and stone (Stones) habitats, use aerial respiration by attaching to vegetation (aerial/vegetation) and are considered deposit feeders that feed on a variety of algae and detritus (Deposit feeder 2; Table S3). Lake Shokwe (Sh LF and Sh HF) and Lake Nyamithi sampled in 2018 (Nya 18) grouped together on axis 1, with aquatic invertebrate traits associated with these sites including those that are considered free‐living (Free‐living), use aerial active dispersal mechanisms (Aerial active) and make use of a spiracle to breath (Aerial: spiracle). Along axis 2 (explaining 24.48% of data variation) separation was largely a result of the Usuthu River separating from the other sampling sites, particularly FL 1 and FL 4. Aquatic invertebrate traits associated with the Usuthu River consisted of those with a high sensitivity to changes in water quality (High sensitivity), that prefer to live in sandy substrates (Sand) and prefer habitats with rapids (Rapids) and are considered deposit feeders that feed on various fine organic particulate organic matter, detritus and algae (Deposit feeder 3). Aquatic invertebrate traits associated with FL 1 and FL 4 included those that prefer living in pools, vegetated habitats (Vegetation) and mud, are scrapers that feed on films of micro‐organisms from substrates (Scraper) and have a plastron (Plastron) or lungs (Aerial: lungs) for breathing.

## 4 DISCUSSION

The aim of this study was to assess the differences in aquatic invertebrate community diversity between a regulated and unregulated river, along with their respective floodplains. Additionally, it aimed to determine whether a lake receiving water from both rivers would resemble the invertebrate communities of either river. Despite their close proximity, the aquatic invertebrate community structures, diversity of taxa, species sensitivities and traits of the Usuthu and Phongolo rivers and their associated floodplains differed greatly from one another.

Although it has a slightly greater diversity of aquatic invertebrates than the Usuthu River, the Phongolo River had fewer taxa that were sensitive to changes in water quality and flow. Although Lake Nyamithi shared a slightly higher number of taxa with the Phongolo River than with the Usuthu River, the invertebrate communities of Lake Nyamithi did not resemble those of either river or its associated floodplain wetlands. All systems also separated from each other in their aquatic invertebrate traits and, in many cases, reflected the traits pattern of the ecosystem they were most associated with. Due to the unique characteristics of Lake Nyamithi, being a naturally saline lake and the only one of its kind in the study region, direct comparisons with other wetlands were difficult to make.

Water quality in the Phongolo River has deteriorated since the 1970s and the construction of the Pongolapoort Dam. In a study of the water quality of the Phongolo River between 1970 and 2017, de Necker, Neswiswi, et al. (2020) determined that the water quality of this river has deteriorated from an unmodified, natural river to a largely modified river as a result of increased salinity and nutrient inputs caused by the Pongolapoort Dam and greater anthropogenic use of the ecosystem. Along with a deterioration of water quality there has been a reduction in flow in recent years resulting from not only increased water extraction for crop irrigation in the surrounding area, but supraseasonal droughts that occurred from 2015 to 2016 (de Necker et al., 2021; de Necker, Neswiswi, et al., 2020; van Rooyen et al., 2022). These factors have impacted the aquatic invertebrate community of the Phongolo River, particularly compared with the less affected and unregulated Usuthu River. This was evident from the types and abundances of aquatic invertebrates found in each river system.

Although the current study recorded a higher diversity of aquatic invertebrates in the Phongolo River compared to the Usuthu River, the overall sensitivity of aquatic invertebrates in the Usuthu River was higher, more closely reflecting the state of the Phongolo River in 2012/2013 when the PRF was still receiving regular flood releases from the Pongolapoort Dam (see Smit et al., 2016). In particular, several rheophilous macroinvertebrate families and those sensitive to changes in water chemistry, previously documented in the Phongolo River (e.g., Ephemeridae and Tricorythidae; see Smit et al., 2016), were absent from this river following the 2015–2016 drought period and have not been recorded since. Instead, a greater abundance of limnophilic taxa typically associated with lentic systems, slow‐flowing water, and with a tolerance for reduced water quality were observed in the Phongolo River during the current study following the drought, reflective of the reduced base flows. This may be attributed to the increased habitat suitability for these particular taxa (Calapez et al., 2017) and include Chydoridae, Corixidae, Culicidae, Libellulidae and Lumbriculidae (Chessman, 2015; Dallas & Day, 2007; Fry, 2021; Gerber & Gabriel, 2002). Similarly, Leigh (2013) reported shifts in aquatic invertebrate diversity in rivers of northern Australia from the wetter to the drier period, as a result of replacement of rheophilous and sensitive taxa with those more adapted to slow‐flowing, and polluted waters.

A direct assessment of flow impact between high flow and low flow was not feasible during this study. However, these fluctuations in flow (i.e., high flow versus low flow) between sampling surveys represent natural, short‐term alterations that aquatic biota are adapted to (de la Fuente et al., 2018; Lytle & Poff, 2004). It is thus unlikely that these naturally occurring differences in flow significantly influenced the observed differences in the macroinvertebrate communities between the Phongolo and Usuthu rivers. Rather, the absence of sensitive macroinvertebrate families from the Phongolo River can be attributed to the long‐term anthropogenic impacts and alterations in habitat, changes in flow velocity and shifts in water chemistry that have occurred on this river since the 1970s. This was then further exacerbated by the drought of 2015–2016 that led to reduced water flow as a result of no flood releases from the Pongolapoort Dam.

Furthermore, the presence of the invasive snail *Physella acuta*, known for its high tolerance to pollutants (Al‐Shami et al., 2011; Fry, 2021), indicates the impact of long‐term alterations in water quality and flow velocity in the Phongolo River. Additionally, several other invasive species, namely the quilted melania (*Tarebia granifera*), water hyacinth (*Eichornia crassipes*), red water fern (*Azolla filiculoides*) and the Australian redclaw crayfish (*Cherax quadricarinuatus*), are also present in this river system (SA Wetlands Conservation Programme, 1996; Smit et al., 2016; van Rensburg, 2016).

In contrast, the Usuthu River exhibited associations with taxa from the families Atyidae, Baetidae, Heptageniidae, Hydropsychidae and Platycnemidae. These taxa are highly flow‐dependant, preferring faster‐flowing water, with Heptageniidae and Baetidae also displaying high sensitivity to reduced water quality (Erasmus et al., 2021; Fry, 2021; Gerber & Gabriel, 2002). Similar observations were reported in a study of regulated and unregulated rivers in Norway, where changes in water quality resulting from river regulation were the primary driver of alterations in aquatic invertebrate community compositions (Schneider & Petrin, 2017). These findings were supported by the presence of aquatic invertebrate traits in the Usuthu River that are more sensitive to changes in water quality and flow. Additionally, only one of the invasive species present in the Phongolo River, the Australian redclaw crayfish, is also known to occur in the Usuthu River (Smit et al., 2016; van Rensburg, 2016), which may also threaten the aquatic biota and water quality of this river system.

It has been widely demonstrated that modifications to flood regimes, including changes in flow velocity and abiotic factors, lead to habitat loss and alterations in the diversity, abundance and biological traits of associated biota (Belmar et al., 2013, 2019; Schneider & Petrin, 2017). The Usuthu and Phongolo rivers also exhibited differences in aquatic invertebrate community structures due to variations in habitat preference and availability. The trait‐based analysis indicated the presence of more sensitive biota with a preference for rapids and sandy habitats in the Usuthu River, while invertebrates that were more tolerant to environmental stressors and preferred riffles and stones were present in the Phongolo River. Habitat preference influences the composition of aquatic invertebrate communities, as many species respond to changes in habitat characteristics, with habitat quality being equally important as water quality (Beauger et al., 2006; Carter et al., 2017). The Usuthu River substrate mainly consists of sand and larger sediment particles, with little to no mud, stones, and in‐stream vegetation, whereas the Phongolo River has a muddier substrate with riffles and in‐stream vegetation (Smit et al., 2016).

The aquatic invertebrate diversity differed greatly between the Usuthu‐associated floodplain wetland (Lake Shokwe) and the Phongolo River floodplain wetlands (FL 1 and FL 4). The Phongolo River floodplain wetlands exhibited a higher number of taxa and diversity compared to Lake Shokwe. This may be attributed to the recent controlled flooding (prior to sampling) from the Pongolapoort Dam in the Phongolo River system, resulting in a large inflow of fresh water and increased connectivity for exchange of aquatic biota, thus increasing aquatic invertebrate taxon richness in the floodplain wetlands. Flooding in a river creates hydrological connectivity between a river and its associated floodplain and increases aquatic invertebrate taxon richness in the associated floodplain wetlands (Dube et al., 2017; Turić et al., 2015). Similar observations were made by Dube et al. (2017) in a previous study of the floodplain wetlands of the Phongolo River and discerned that aquatic invertebrate richness increased immediately after flood releases compared to when the river and its floodplain are disconnected. This was also found by Gallardo et al. (2008) during a study of a regulated river floodplain in Spain.

The trait‐based analysis of aquatic invertebrates further confirmed the distinct differences in habitat between the floodplain wetlands. The Phongolo River floodplain wetlands featured muddy substrates and abundant aquatic floating vegetation (Dube et al., 2017), while Lake Shokwe had minimal aquatic vegetation, mainly consisting of marginal vegetation (van Rooyen et al., 2022; Whittington et al., 2013). These differences in physical habitat likely contributed to variations in aquatic invertebrate diversity, as greater availability of habitat structures positively correlates with high biodiversity (Brendonck et al., 2022; Collier et al., 2016; Dube et al., 2022; Piedade et al., 2022). Furthermore, the aquatic invertebrates in these floodplain systems showed adaptations to low oxygen conditions, using spiracles or plastrons for air breathing. Taxa capable of breathing atmospheric air tend to dominate stagnant habitats with low oxygen, which is common in wetland ecosystems, particularly during low‐flow or natural drought periods (Batzer & Boix, 2016; Deemy et al., 2022; Reichard, 2022).

The Usuthu River is reportedly an important water source for both Lake Shokwe and Lake Nyamithi (de Necker et al., 2021; van Rooyen et al., 2022). It was therefore initially expected that Lake Nyamithi would have a similar aquatic invertebrate community composition to that of the Usuthu River, as the lake was sampled when its primary water source was this river, and also because van Rooyen et al. (2022) found the water quality and metal concentrations of Lake Nyamithi to be more similar to the Usuthu River than that of the Phongolo River. However, our findings revealed large differences in the aquatic invertebrate communities of Lake Nyamithi compared to both rivers, with over 80% dissimilarity. This can be attributed to the unique ecological characteristics of Lake Nyamithi as a natural saline lake, experiencing salinity fluctuations due to dry/wet cycles (de Necker et al., 2021; de Necker, Brendonck, et al., 2022). Consequently, the aquatic invertebrates in this lake exhibit distinct adaptations to survive and thrive in such a distinct environment, resulting in a unique diversity of organisms (de Necker et al., 2021; de Necker, Brendonck, et al., 2022). Certain taxa, namely Ostracoda and *Sigara* sp., were exclusive to Lake Nyamithi or were more dominant in this lake compared with the other ecosystems, namely *Berosus* sp., *Bezzia* sp. Chironominae, *Micronecta* sp., Oligochaeta and Tanypodinae. These organisms are known for their ability to tolerate environmental changes and adapt to salinity conditions (de Necker et al., 2021; Velasco et al., 2006; Waterkeyn et al., 2008, 2010). Similarly, in a study of Lake Nyamithi in 2016, de Necker et al. (2021) found that Ostracoda, *Sigara* sp., *Micronecta* and *Berosus* sp. are highly abundant in Lake Nyamithi, resulting from their ability to tolerate high levels of salinity (Brendonck et al., 2022; de Necker et al., 2021; Waterkeyn et al., 2008). In a study of saline lakes in northern Tibet, Wen et al. (2005) also found that the most abundant taxon in saline lakes was Ostracoda (40.9% occurrence) while in Australia Khan (2003) also found Ostracoda, Corixidae (*Sigara* sp. and *Micronecta* sp.), Hydrophilidae (*Berosus* sp.) Oligochaeta and Ceratopogonidae (*Bezzia* sp.) in the four saline lakes sampled.

The presence of *Tarebia granifera*, an extremely invasive species, in Lake Nyamithi is concerning, because of its resistance to salinity, changes in temperature, and drought, providing it a competitive advantage over many native aquatic biota. It can outcompete most aquatic molluscs in an invaded ecosystem and most likely has severe bottom‐up effects on the food web (Miranda et al., 2010; Miranda & Perissinotto, 2014). It should be noted that *T. granifera* has also been reported in reaches of the Phongolo River floodplain wetlands both within and outside the study area (de Necker et al., 2021; Dube et al., 2017).

## 5 CONCLUSION

The present study provided baseline information on the aquatic invertebrate communities of the Usuthu River, an unregulated and largely unexploited river in southern Africa. This river supported a high diversity of sensitive aquatic invertebrates, indicating the high conservation value of this river and its floodplain ecosystem. The first hypothesis for this study stating that there will be no demonstrable differences in the aquatic invertebrate communities between those of more impacted Phongolo River and the less impact Usuthu River, is rejected. Although the Phongolo River has not seen a great loss of aquatic invertebrate diversity in the last decade, it is clearly being affected by the anthropogenic use and river regulation, particularly in comparison to an unregulated river such as the Usuthu River. Despite the general greater diversity of aquatic invertebrates in the Phongolo River, the taxa inhabiting it were generally more resilient to changes in water quality and flow velocity, compared to those found in the Usuthu River. If mismanagement of flooding from the Pongolapoort Dam and overuse by humans continues, it could lead to the total loss of sensitive aquatic invertebrate communities in the Phongolo River. Moreover, the occurrence of invasive molluscan species such as *P. acuta* and *T. granifera* in the Phongolo River and Lake Nyamithi presents a significant threat to not only the aquatic invertebrate community, but also other aquatic biota within the system. Notably, these invasive species are currently absent in the Usuthu River. However, it is noteworthy to mention that the Australian redclaw crayfish is present in both river systems, placing both ecosystems, particularly the less impacted Usuthu River, under threat. The present study also highlights the importance of ensuring adequate water flow in rivers and floodplain wetlands to support high biodiversity and sensitive taxa. This was demonstrated by the higher diversity of aquatic invertebrates in the floodplain wetlands associated with the Phongolo River compared with Lake Shokwe, that had recently been connected to the Phongolo River which led to the inflow of freshwater and thus a higher aquatic invertebrate diversity. The second hypothesis stating the aquatic invertebrate communities of Lake Nyamithi would resemble the Usuthu River more closely than the Phongolo River, is rejected. The aquatic invertebrate community of Lake Nyamithi are a composition of taxa adapted to the unique alterations in environmental conditions that occur within this system and highlights the ecological value in of protecting unique ecosystems such as this.

## AUTHOR CONTRIBUTIONS

**Lizaan de Necker:** Conceptualization (equal); data curation (supporting); formal analysis (lead); investigation (equal); writing – original draft (lead); writing – review and editing (lead). **Divan van Rooyen:** Conceptualization (equal); data curation (lead); formal analysis (supporting); investigation (equal); methodology (equal); writing – original draft (supporting); writing – review and editing (supporting). **Ruan Gerber:** Conceptualization (equal); data curation (supporting); formal analysis (supporting); investigation (supporting); methodology (equal); project administration (supporting); supervision (equal); writing – original draft (supporting); writing – review and editing (supporting). **Luc Brendonck:** Funding acquisition (equal); project administration (equal); supervision (equal); writing – review and editing (supporting). **Victor Wepener:** Funding acquisition (equal); project administration (equal); resources (equal); supervision (equal); writing – review and editing (supporting). **Nico J. Smit:** Funding acquisition (equal); project administration (supporting); resources (equal); supervision (equal); writing – review and editing (supporting).

## CONFLICT OF INTEREST STATEMENT

The authors have no conflict of interest to declare.

### OPEN RESEARCH BADGES

This article has earned an Open Data badge for making publicly available the digitally‐shareable data necessary to reproduce the reported results. The data is available at https://doi.org/10.5061/dryad.12jm63z4d.

## Supporting information


Data S1.

## Data Availability

The data that support the findings of this study are openly available in the Dryad repository at https://doi.org/10.5061/dryad.12jm63z4d.
