# Supplementary material for: Effects of river regulation on aquatic invertebrate community composition: A comparative analysis in two southern African rivers
Source: Ecol Evol. 2024 Feb 7;14(2):e10963. doi: 10.1002/ece3.10963 (PMC10847884; doi:10.1002/ece3.10963)
Supplement: Supplementary file 1 — Data S1. [file ECE3-14-e10963-s001.docx]

Supplementary Tables

Table S1 Aquatic invertebrate traits associated with taxa collected in all sites during the current and previous surveys. – indicates no data available.

| **Species** | **Respiration** | **Habitat preference** | **Hydraulic preference** | **Dispersal** | **FFG** | **Sensitivity** |
| --- | --- | --- | --- | --- | --- | --- |
| *Afrocaenis* sp. | Gills | Stones | – | – | Deposit feeder 3 | Moderately tolerant |
| *Afrogyrus coretus* | Aerial: lungs | Vegetation | Indifferent | – | – | Highly tolerant |
| *Afronurus* sp. | Gills | Stones | Riffles | Aerial active | Grazer | Highly sensitive |
| *Agraptocorixa* sp. | Aerial: spiracle | Free living | Indifferent | – | Predator | Highly tolerant |
| *Ahaggaria australis* | Plastron | Stones | Riffles | Aquatic active | – | Moderately sensitive |
| *Allochemis leucosticta* | Gills | Vegetation | Pools | – | Predator | Moderately tolerant |
| *Allocotocerus* sp. | Plastron | Vegetation | – | Aerial active | Scraper | Moderately tolerant |
| *Anaciaeschna* sp. | Gills | – | Pools | Aquatic active | Predator | Moderately sensitive |
| *Anax* sp. | Gills | Free living | Pools | Aquatic active | Predator | Moderately sensitive |
| *Angila* sp. | Aerial: spiracle | Vegetation | Pools | Aerial active | Predator | Moderately tolerant |
| *Anisops* sp. | Aerial: spiracle | Vegetation | Pools | Aerial active | Predator | Highly tolerant |
| *Anopheles* sp. | Aerial/vegetation | Vegetation | Indifferent | – | Filter feeder | Highly tolerant |
| *Appasus* sp. | Aerial: spiracle | Vegetation | Pools | – | Predator | Highly tolerant |
| *Aulonogyrus* sp. | Aerial: spiracle | Free living | Indifferent | Aquatic active | Predator | Moderately tolerant |
| Baetidae | Gills | Indifferent | Indifferent | Aquatic active | Deposit feeder 1 | Moderately tolerant |
| *Berosus* sp. | Plastron | Vegetation | Temporary pools | Aerial active | Scraper | Moderately tolerant |
| *Bezzia* sp. | Gills | Mud | Indifferent |  | Grazer | Moderately tolerant |
| *Brachythemis leucosticta* | Gills | Mud | Pools | Aquatic active | Predator | Moderately tolerant |
| *Bradinopyga cornuta* | Gills | Stones | Pools | Aquatic active | Predator | Moderately tolerant |
| *Bulinus depressus* | – | – | – | – | Scraper | Highly tolerant |
| *Bulinus forskaii* | – | – | – | – | Scraper | Highly tolerant |
| *Bulinus natalensis* | – | – | – | – | Scraper | Highly tolerant |
| *Bulinus tropicus* | – | – | – | – | Scraper | Highly tolerant |
| *Caenis* sp. | Gills | Gravel, sand and mud | – | – | Deposit feeder 3 | Moderately tolerant |
| *Canthydrus* sp. | – | Mud | Pools | Aquatic active | Predator | Moderately tolerant |
| *Caridina nilotica* | Gills | Gravel, sand and mud | Indifferent | Aquatic active | Deposit feeder 2 | Moderately sensitive |
| *Ceratogomphus* sp. | Gills | Gravel, sand and mud | Pools | Aquatic passive | Predator | Moderately tolerant |
| *Ceriagrion* sp. | Gills | Vegetation | Pools | – | Predator | Moderately tolerant |
| Chironominae | Gills | Indifferent | Pools | Aquatic active | Deposit feeder 1 | Highly tolerant |

**Table S1 continued**

| **Species** | **Respiration** | **Habitat preference** | **Hydraulic preference** | **Dispersal** | **FFG** | **Sensitivity** |
| --- | --- | --- | --- | --- | --- | --- |
| Chydoridae | Gills | Mud | Bottom dwelling | – | Filter feeder | – |
| *Cloeon* and *Procloeon* sp. | Gills | Vegetation | Riffles | Aquatic active | Deposit feeder 2 | Moderately tolerant |
| *Clogmia albopunctata* | Aerial/vegetation | Indifferent | – | – | Deposit feeder 2 | Highly tolerant |
| Coenagrionidae | Gills | Gravel, sand and mud | – | – | Predator | Moderately tolerant |
| *Corbicula fluminalis* | – | Gravel, sand and mud | Indifferent | – | Filter feeder | Moderately tolerant |
| Crambidae | – | Vegetation | Pools | Aerial active | Shredder | Highly sensitive |
| *Culex* sp. | Aerial/vegetation | Stones | Indifferent | – | Filter feeder | Highly tolerant |
| *Culicoides* sp. | Gills | Mud | Indifferent | – | Filter feeder | Highly tolerant |
| Curculionidae | Plastron | Vegetation | Indifferent | Aquatic active | Grazer | – |
| *Cybister* sp. | Plastron | Free living | Pools | Aquatic active | Predator | Moderately tolerant |
| Cyprididae | – | – | – | – | – | – |
| Darwinulidae | Tegument/Cutaneous | Vegetation | Pools | – | – | – |
| *Dineutus* sp. | Aerial: spiracle | Free living | Indifferent | Aquatic active | Predator | Moderately tolerant |
| Dolichopodidae | Aerial: spiracle | – | – | – | Predator | – |
| Dytiscidae | Plastron | Free living | Pools | – | Predator | Moderately tolerant |
| Elmidae | Plastron | – | – | – | – | – |
| Empididae | – | Stones | Indifferent | – | Predator | Moderately tolerant |
| *Enallagma* sp. | Gills | Stones | – | Aquatic passive | Predator | Moderately tolerant |
| *Enithares* sp. | Aerial: spiracle | Free living | Pools | Aerial active | Predator | Highly tolerant |
| *Enochrus* sp. | Plastron | Vegetation | Pools | Aerial active | Scraper | Moderately tolerant |
| *Ephoron* sp. | Gills | Sand | – | – | – | – |
| *Eurymetra* sp. | Aerial: spiracle | Vegetation | Pools | Aerial active | Predator | Moderately tolerant |
| *Euthraulus* sp. | Gills | Stones | Pools |  | Deposit feeder 1 | Moderately sensitive |
| *Gerris* sp. | Aerial: spiracle | Vegetation | Pools | Aerial active | Predator | Moderately tolerant |
| Glossosomatidae | – | Stones | – | – | Scraper | Moderately sensitive |
| *Gyraulus costulatus* | – | Stones | Indifferent | – | Scraper | Highly tolerant |
| *Helochares* sp. | Plastron | Vegetation | Pools | Aerial active | Scraper | Moderately tolerant |
| Hirudinea | Tegument/Cutaneous |  | Pools | – | Predator | Highly tolerant |
| *Hydaticus* sp. | Plastron | Free living | Pools | Aquatic active | Predator | Moderately tolerant |

**Table S1 continued**

| **Species** | **Respiration** | **Habitat preference** | **Hydraulic preference** | **Dispersal** | **FFG** | **Sensitivity** |
| --- | --- | --- | --- | --- | --- | --- |
| Hydrachnellae | – | – | – | – | Predator | – |
| *Hydrocanthus* sp. | Plastron | Vegetation | Pools | Aquatic active | Predator | Moderately tolerant |
| *Hydroglyphus* sp. | Plastron | Free living | Pools | Aquatic active | Predator | Moderately tolerant |
| *Hydrometra* sp. | Aerial: spiracle | Vegetation | Pools | Aerial active | Predator | Moderately tolerant |
| *Hydrophilus* sp. | Plastron | Vegetation | Pools | – | Scraper | Moderately tolerant |
| *Hydropsyche* sp. | Gills | Stones | Rapid | – | Predator | Moderately tolerant |
| *Hydrovatus* sp. | Plastron | Vegetation | Pools | Aquatic active | Predator | Moderately tolerant |
| *Hyphydrus* sp. | Plastron | Free living | Pools | Aquatic active | Predator | Moderately tolerant |
| *Ictinogomphus ferox* | Gills | Mud | Pools | Aquatic passive | Predator | Moderately tolerant |
| *Laccobius* sp. | Plastron | Vegetation | Pools | Aerial active | Scraper | Moderately tolerant |
| *Laccocoris* sp. | Aerial: spiracle | Vegetation | Pools | Aerial active | Predator | Moderately sensitive |
| *Laccophilus* sp. | Plastron | Free living | Pools | Aquatic active | Predator | Moderately tolerant |
| *Laccotrephes* sp. | Aerial/vegetation | Vegetation | Pools | Aerial active | Predator | Highly tolerant |
| Larainae | – | – | – | – | – | – |
| *Leptelmis* sp. | Plastron | Stones | Riffles | Aquatic active | Scraper | Moderately sensitive |
| Leptoceridae | – | Indifferent | – | – | Omnivore | Moderately tolerant |
| *Lethocerus niloticus* | Aerial: spiracle | Vegetation | Pools | Aerial active | Predator | Highly tolerant |
| *Limnogeton fieberi* | Aerial: spiracle | Vegetation | Pools | Aerial active | Predator | Highly tolerant |
| *Limnogonus* sp. | Aerial: spiracle | Vegetation | Pools | Aerial active | Predator | Moderately tolerant |
| *Limonia* sp. | Gills | Vegetation | Indifferent | – | Predator | Moderately tolerant |
| Lumbriculidae | Tegument/Cutaneous | – | – | – | Deposit feeder 1 | Highly tolerant |
| *Lymnaea natalensis* | – | Vegetation | Indifferent | – | Scraper | Highly tolerant |
| *Macrocoris* sp. | Aerial: spiracle | Vegetation | Pools | Aerial active | Predator | Moderately sensitive |
| *Mansonia* sp. | Aerial/vegetation | Indifferent | Indifferent | – | Filter feeder | Highly tolerant |
| *Melanoides tuberculata* | – | Stones | Indifferent | – | Scraper | Highly tolerant |
| *Mesovelia* sp. | Aerial: spiracle | Vegetation | Pools | Aerial active | Predator | Moderately tolerant |
| *Microgomphus mozambicensis* | Gills | Mud | Pools | Aquatic passive | Predator | Moderately tolerant |
| *Micronecta* sp. | Aerial: spiracle | Free living | Indifferent | Aerial active | Predator | Highly tolerant |
| *Moina micrura* | – | – | – | – | – | – |

**Table S1 continued**

| **Species** | **Respiration** | **Habitat preference** | **Hydraulic preference** | **Dispersal** | **FFG** | **Sensitivity** |
| --- | --- | --- | --- | --- | --- | --- |
| *Naboandelus africanus* | Aerial: spiracle | Vegetation | Pools | Aerial active | Predator | Moderately tolerant |
| Naididae | Tegument/Cutaneous | – | – | – | Grazer | Highly tolerant |
| *Naucoris* sp. | Aerial: spiracle | Vegetation | Pools | Aerial active | Predator | Moderately sensitive |
| *Neogerris* sp. | Aerial: spiracle | Vegetation | Pools | – | Predator | Moderately tolerant |
| *Neohydrocoptus* sp. | Plastron | Mud | Pools | Aquatic active | Predator | Moderately tolerant |
| *Neomacrocoris* sp. | Aerial: spiracle | Vegetation | Pools | Aerial active | Predator | Moderately sensitive |
| *Neumania* sp. | – | – | – | – | – | – |
| *Nilus margartatus* | – | – | – | – | – | – |
| *Nychia limpida* | Aerial: spiracle | Free living | Pools | Aerial active | Predator | Highly tolerant |
| Oligochaeta | – | – | – | – | – | Highly tolerant |
| *Olpogastra* sp. | Gills | Vegetation | Pools | – | Predator | Moderately tolerant |
| *Orectogyrus* sp. | Aerial: spiracle | Free living | Indifferent | Aquatic active | Predator | Moderately tolerant |
| *Orthetrum* sp. | Gills | Mud | Pools | Aquatic active | Predator | Moderately tolerant |
| Orthocladiinae | Gills | Stones | Runs | Aquatic active | Scraper | Highly tolerant |
| Ostracoda | – | – | – | – | Deposit feeder 1 | – |
| *Pantala flavescens* | Gills | Vegetation | Pools | Aquatic active | Predator | Moderately tolerant |
| *Paragomphus* sp. | Gills | Mud | Pools | Aquatic passive | Predator | Moderately tolerant |
| *Parasthetops* sp. | Aerial: spiracle | Stones | Indifferent |  | Scraper | Moderately sensitive |
| *Phaon iridipennis* | Gills | Vegetation | Riffles | Aquatic passive | Predator | Moderately sensitive |
| *Physella acuta* | – | Vegetation | – | – | – | Highly tolerant |
| *Pirata* sp. | – | – | – | – | – | – |
| *Plea* sp. | Aerial: spiracle | Vegetation | Pools | Aerial active | Predator | Moderately tolerant |
| *Pseudagrion* sp. | Gills | Vegetation | Indifferent | – | Predator | Moderately tolerant |
| *Ranatra* sp. | Aerial/vegetation | Vegetation | Pools | – | Predator | Highly tolerant |
| *Regimbartia* sp. | Plastron | Vegetation | – | Aerial active | Scraper | Moderately tolerant |
| *Rhagadotarsus hutchinsonii* | Aerial: spiracle | Vegetation | Pools | Aerial active | Predator | Moderately tolerant |
| *Rhagovelia* sp. | Aerial: spiracle | Free living | Pools | Aerial active | Predator | Moderately tolerant |
| *Rhantus* sp. | Plastron | Vegetation | Pools | – | Predator | Moderately tolerant |
| *Rhyssemus* sp. | – | – | – | – | – | – |

**Table S1 continued**

| **Species** | **Respiration** | **Habitat preference** | **Hydraulic preference** | **Dispersal** | **FFG** | **Sensitivity** |
| --- | --- | --- | --- | --- | --- | --- |
| *Sigara* sp. | Aerial: spiracle | Free living | Indifferent | Aerial active | Predator | Highly tolerant |
| *Simocephalus serrulatus* | – | – | Bottom dwelling | – | Filter feeder | – |
| *Simulium* sp. | Gills | Stones | Indifferent | – | Filter feeder | Moderately tolerant |
| *Spercheus* sp. | Plastron | Free living | Pools | – | Filter feeder | – |
| Syrphidae | Aerial/vegetation | Gravel, sand and mud | Indifferent | – | Filter feeder | Highly tolerant |
| Tabanidae | Aerial: spiracle | Stones | Indifferent | – | Predator | Moderately tolerant |
| *Tabanus* sp. | Aerial: spiracle | Mud | Indifferent | – | Predator | Moderately tolerant |
| Tanypodinae | Gills | Indifferent | – | Aquatic active | Predator | Highly tolerant |
| *Tarebia granifera* | – | – | – | – | Scraper | Highly tolerant |
| *Tetragnatha* sp. | – | – | – | – | – | – |
| *Tetrathemis polleni* | Gills | Stones | Pools | Aquatic active | Predator | Moderately tolerant |
| *Thermocyclops* sp. | Tegument/Cutaneous | – | Indifferent | Aquatic active | Predator | – |
| *Tipula* sp. | – | Mud | Indifferent | – | Predator | Moderately tolerant |
| *Tricorythus* sp. | Gills | Stones | – | – | Deposit feeder 2 | – |
| Unionicolidae | Gills | Gravel, sand and mud | Indifferent | – | – | Moderately tolerant |

Table S2: Detailed list of aquatic invertebrates and families collected during the present study in the Usuthu River (US 18), Lake Shokwe during the low flow (Sh LF) and high flow (Sh HF) and Lake Nyamithi (Nyamithi 18) as well as the Phongolo River (Ph 16), its associated floodplain lakes (FL 1 and FL 4) and Lake Nyamithi (Nyamithi 17) sampled by Dube et al., (2017), de Necker (2019) and de Necker et al. (2021).

| **Family** | **Species** | **Us 18** | **Sh LF** | **Sh HF** | **Nyamithi 18** | **Ph 16** | **FL 1** | **FL 4** | **Nyamithi 17** |
| --- | --- | --- | --- | --- | --- | --- | --- | --- | --- |
| Aeshnidae | *Anaciaeschna* sp. | 0 | 0 | 4 | 0 | 0 | 7 | 1 | 0 |
| Aeshnidae | *Anax* sp. | 0 | 0 | 12 | 0 | 4 | 0 | 14 | 0 |
| Atyidae | *Caridina nilotica* | 114 | 0 | 3 | 4 | 12 | 12 | 7 | 34 |
| Baetidae | Baetidae | 296 | 0 | 151 | 38 | 2 | 0 | 0 | 0 |
| Baetidae | *Cloeon* and *Procloeon* sp. | 0 | 0 | 0 | 0 | 5 | 18 | 39 | 0 |
| Belostomatidae | *Appasus* sp. | 20 | 0 | 0 | 0 | 0 | 18 | 29 | 16 |
| Belostomatidae | *Lethocerus niloticus* | 0 | 0 | 0 | 0 | 0 | 14 | 8 | 0 |
| Belostomatidae | *Limnogeton fieberi* | 1 | 0 | 0 | 0 | 0 | 0 | 0 | 0 |
| Caenidae | *Afrocaenis* sp. | 31 | 0 | 0 | 0 | 0 | 0 | 0 | 0 |
| Caenidae | *Caenis* sp. | 70 | 0 | 0 | 6 | 0 | 0 | 0 | 0 |
| Caloptegyridae | *Phaon iridipennis* | 3 | 0 | 0 | 0 | 0 | 0 | 0 | 0 |
| Ceratopogonidae | *Bezzia* sp. | 0 | 0 | 1 | 164 | 0 | 0 | 0 | 3 |
| Ceratopogonidae | *Culicoides* sp. | 0 | 0 | 2 | 0 | 0 | 0 | 0 | 0 |
| Chironomidae | Chironominae | 0 | 0 | 2 | 1 | 83 | 6 | 4 | 20 |
| Chironomidae | Orthocladiinae | 0 | 0 | 0 | 0 | 0 | 68 | 0 | 0 |
| Chydoridae | Chydoridae | 0 | 0 | 0 | 0 | 27 | 0 | 0 | 0 |
| Coenagrionidae | *Ceriagrion* sp. | 0 | 0 | 2 | 3 | 0 | 0 | 0 | 0 |
| Coenagrionidae | Coenagrionidae | 0 | 0 | 0 | 0 | 0 | 0 | 0 | 1 |
| Coenagrionidae | *Enallagma* sp. | 0 | 0 | 0 | 0 | 0 | 7 | 2 | 3 |
| Coenagrionidae | *Pseudagrion* sp. | 0 | 0 | 21 | 0 | 17 | 0 | 0 | 10 |
| Corbiculidae | *Corbicula fluminalis* | 0 | 0 | 0 | 2 | 10 | 0 | 0 | 0 |
| Corixidae | *Agraptocorixa* sp. | 0 | 0 | 214 | 39 | 0 | 16 | 1 | 0 |
| Corixidae | *Micronecta* sp. | 3 | 362 | 482 | 869 | 1 | 0 | 0 | 10 |
| Corixidae | *Sigara* sp. | 0 | 0 | 0 | 0 | 0 | 0 | 0 | 15 |
| Crambidae | Crambidae | 0 | 0 | 9 | 1 | 1 | 0 | 0 | 0 |
| Culicidae | *Anopheles* sp. | 1 | 0 | 6 | 1 | 0 | 0 | 0 | 0 |
| Culicidae | *Culex* sp. | 0 | 0 | 2 | 1 | 12 | 0 | 0 | 0 |
| Culicidae | *Mansonia* sp. | 0 | 0 | 0 | 0 | 1 | 0 | 0 | 0 |
| Curculionidae | Curculionidae | 0 | 0 | 0 | 0 | 0 | 0 | 0 | 1 |
| Cyclopidae | *Thermocyclops* sp. | 0 | 0 | 0 | 0 | 47 | 0 | 0 | 0 |
| Cyprididae | Cyprididae | 0 | 0 | 0 | 0 | 7 | 0 | 0 | 0 |
| Daphniidae | *Simocephalus serrulatus* | 0 | 0 | 0 | 0 | 11 | 0 | 0 | 0 |

**Table S2 continued**

| **Family** | **Species** | **Us 18** | **Sh LF** | **Sh HF** | **Nyamithi 18** | **Ph 16** | **FL 1** | **FL 4** | **Nyamithi 17** |
| --- | --- | --- | --- | --- | --- | --- | --- | --- | --- |
| Darwinulidae | Darwinulidae | 0 | 0 | 0 | 0 | 7 | 0 | 0 | 0 |
| Dolichopodidae | Dolichopodidae | 0 | 0 | 0 | 0 | 0 | 0 | 2 | 0 |
| Dryopidae | *Ahaggaria australis* | 0 | 0 | 0 | 0 | 0 | 0 | 4 | 0 |
| Dytiscidae | *Cybister* sp. | 0 | 1 | 3 | 0 | 4 | 0 | 0 | 4 |
| Dytiscidae | Dytiscidae | 0 | 0 | 0 | 0 | 1 | 1 | 5 | 0 |
| Dytiscidae | *Hydaticus* sp. | 0 | 3 | 3 | 0 | 0 | 0 | 0 | 3 |
| Dytiscidae | *Hydroglyphus* sp. | 0 | 1 | 0 | 0 | 0 | 0 | 0 | 1 |
| Dytiscidae | *Hydrovatus* sp. | 0 | 0 | 1 | 0 | 0 | 0 | 0 | 0 |
| Dytiscidae | *Hyphydrus*  sp. | 0 | 0 | 0 | 22 | 0 | 6 | 16 | 34 |
| Dytiscidae | *Laccophilus* sp. | 0 | 0 | 0 | 0 | 0 | 6 | 19 | 3 |
| Dytiscidae | *Rhantus* sp. | 0 | 0 | 1 | 0 | 0 | 0 | 0 | 0 |
| Elmidae | Elmidae | 0 | 0 | 0 | 0 | 1 | 0 | 0 | 0 |
| Elmidae | Larainae | 1 | 1 | 1 | 0 | 0 | 0 | 0 | 0 |
| Elmidae | *Leptelmis* sp. | 1 | 0 | 0 | 0 | 0 | 0 | 0 | 0 |
| Empididae | Empididae | 0 | 1 | 0 | 0 | 0 | 0 | 0 | 0 |
| Gerridae | *Eurymetra* sp. | 0 | 0 | 4 | 0 | 0 | 0 | 0 | 0 |
| Gerridae | *Gerris* sp. | 0 | 0 | 3 | 0 | 0 | 0 | 0 | 0 |
| Gerridae | *Limnogonus* sp. | 0 | 0 | 0 | 2 | 0 | 0 | 0 | 0 |
| Gerridae | *Naboandelus africanus* | 0 | 0 | 0 | 1 | 0 | 0 | 0 | 0 |
| Gerridae | *Neogerris* sp. | 0 | 0 | 8 | 0 | 0 | 0 | 22 | 0 |
| Gerridae | *Rhagadotarsus hutchinsonii* | 0 | 0 | 0 | 31 | 0 | 0 | 0 | 0 |
| Glossomatidae | Glossosomatidae | 0 | 1 | 0 | 0 | 0 | 0 | 0 | 0 |
| Gomphidae | *Ceratogomphus* sp. | 0 | 0 | 0 | 2 | 0 | 0 | 0 | 0 |
| Gomphidae | *Ictinogomphus ferox* | 0 | 0 | 0 | 0 | 0 | 0 | 0 | 2 |
| Gomphidae | *Microgomphus mozambicensis* | 0 | 0 | 0 | 0 | 1 | 0 | 0 | 0 |
| Gomphidae | *Paragomphus* sp. | 4 | 0 | 0 | 7 | 0 | 0 | 0 | 0 |
| Gyrinidae | *Aulonogyrus* sp. | 118 | 0 | 0 | 0 | 2 | 0 | 0 | 0 |
| Gyrinidae | *Dineutus* sp. | 0 | 2 | 0 | 0 | 0 | 0 | 0 | 0 |
| Gyrinidae | *Orectogyrus* sp. | 0 | 0 | 2 | 0 | 0 | 0 | 0 | 0 |
| Heptageniidae | *Afronurus* sp. | 24 | 0 | 0 | 0 | 0 | 0 | 0 | 0 |
| Hirudinea | Hirudinea | 0 | 1 | 3 | 0 | 0 | 0 | 0 | 0 |
| Hydrachnidae | Hydrachnellae | 0 | 0 | 0 | 0 | 1 | 5 | 9 | 0 |
| Hydraenidae | *Parasthetops* sp. | 1 | 2 | 6 | 0 | 0 | 0 | 0 | 0 |

**Table S2 continued**

| **Family** | **Species** | **Us 18** | **Sh LF** | **Sh HF** | **Nyamithi 18** | **Ph 16** | **FL 1** | **FL 4** | **Nyamithi 17** |
| --- | --- | --- | --- | --- | --- | --- | --- | --- | --- |
| Hydrometridae | *Hydrometra* sp. | 3 | 1 | 0 | 0 | 0 | 0 | 0 | 0 |
| Hydrophilidae | *Allocotocerus* sp. | 0 | 0 | 0 | 0 | 0 | 9 | 0 | 0 |
| Hydrophilidae | *Enochrus* sp. | 0 | 5 | 23 | 4 | 4 | 0 | 9 | 0 |
| Hydrophilidae | *Helochares* sp. | 0 | 0 | 0 | 0 | 0 | 1 | 0 | 0 |
| Hydrophilidae | *Hydrophilus* sp. | 0 | 0 | 10 | 0 | 0 | 8 | 19 | 1 |
| Hydrophilidae | *Laccobius* sp. | 0 | 0 | 6 | 0 | 7 | 0 | 0 | 2 |
| Hydrophilidae | *Regimbartia* sp. | 0 | 0 | 0 | 0 | 0 | 0 | 10 | 0 |
| Hydrophillidae | *Berosus* sp. | 4 | 7 | 32 | 55 | 0 | 16 | 10 | 33 |
| Hydropscychidae | *Hydropsyche* sp. | 7 | 0 | 0 | 0 | 0 | 0 | 0 | 0 |
| Leptoceridae | Leptoceridae | 1 | 0 | 0 | 4 | 0 | 0 | 0 | 0 |
| Leptophlebiidae | *Euthraulus* sp. | 1 | 0 | 0 | 0 | 0 | 0 | 0 | 0 |
| Libellulidae | *Brachythemis leucosticta* | 0 | 0 | 0 | 0 | 0 | 0 | 0 | 27 |
| Libellulidae | *Bradinopyga cornuta* | 0 | 0 | 0 | 0 | 1 | 0 | 0 | 0 |
| Libellulidae | *Olpogastra* sp. | 0 | 0 | 0 | 0 | 1 | 0 | 0 | 0 |
| Libellulidae | *Orthetrum* sp. | 0 | 0 | 0 | 0 | 3 | 0 | 0 | 0 |
| Libellulidae | *Pantala flavescens* | 0 | 0 | 3 | 7 | 0 | 1 | 0 | 0 |
| Libellulidae | *Tetrathemis polleni* | 0 | 0 | 0 | 0 | 17 | 0 | 0 | 0 |
| Lumbriculidae | Lumbriculidae | 0 | 0 | 0 | 0 | 1 | 0 | 0 | 2 |
| Lycosidae | *Pirata* sp. | 0 | 8 | 10 | 0 | 0 | 0 | 0 | 0 |
| Lymnaeidae | *Lymnaea natalensis* | 0 | 0 | 0 | 0 | 3 | 0 | 0 | 0 |
| Moiniidae | *Moina micrura* | 1 | 0 | 1 | 0 | 0 | 0 | 0 | 0 |
| Naididae | Naididae | 0 | 0 | 0 | 0 | 1 | 0 | 0 | 0 |
| Naucoridae | *Laccocoris* sp. | 8 | 0 | 0 | 1 | 0 | 0 | 0 | 13 |
| Naucoridae | *Macrocoris* sp. | 0 | 0 | 0 | 6 | 0 | 0 | 0 | 0 |
| Naucoridae | *Naucoris* sp. | 0 | 1 | 0 | 0 | 0 | 0 | 0 | 0 |
| Naucoridae | *Neomacrocoris* sp. | 0 | 0 | 5 | 0 | 0 | 0 | 0 | 0 |
| Nepidae | *Laccotrephes* sp. | 1 | 0 | 0 | 3 | 0 | 0 | 0 | 0 |
| Nepidae | *Ranatra* sp. | 0 | 0 | 0 | 0 | 1 | 2 | 5 | 1 |
| Noteridae | *Canthydrus* sp. | 0 | 0 | 0 | 0 | 0 | 4 | 7 | 4 |
| Noteridae | *Hydrocanthus* sp. | 0 | 0 | 1 | 0 | 0 | 0 | 0 | 0 |
| Noteridae | *Neohydrocoptus* sp. | 0 | 0 | 0 | 0 | 0 | 2 | 31 | 0 |
| Noteridae | *Nychia limpida* | 0 | 1 | 0 | 0 | 0 | 0 | 0 | 25 |
| Notonectidae | *Anisops* sp. | 4 | 14 | 26 | 148 | 14 | 73 | 101 | 21 |

**Table S2 continued**

| **Family** | **Species** | **Us 18** | **Sh LF** | **Sh HF** | **Nyamithi 18** | **Ph 16** | **FL 1** | **FL 4** | **Nyamithi 17** |
| --- | --- | --- | --- | --- | --- | --- | --- | --- | --- |
| Notonectidae | *Enithares* sp. | 1 | 1 | 89 | 57 | 0 | 1 | 0 | 0 |
| Oligochaeta | Oligochaeta | 5 | 13 | 56 | 119 | 0 | 35 | 0 | 0 |
| Ostracoda | Ostracoda | 0 | 0 | 0 | 565 | 0 | 0 | 0 | 46 |
| Physidae | *Physella acuta* | 0 | 0 | 0 | 0 | 11 | 0 | 0 | 0 |
| Pisauridae | *Nilus margartatus* | 10 | 1 | 1 | 0 | 0 | 0 | 0 | 0 |
| Planorbidae | *Afrogyrus coretus* | 0 | 0 | 0 | 0 | 0 | 1 | 2 | 0 |
| Planorbidae | *Bulinus depressus* | 0 | 1 | 0 | 0 | 0 | 1 | 0 | 1 |
| Planorbidae | *Bulinus forskaii* | 0 | 0 | 0 | 0 | 0 | 4 | 27 | 0 |
| Planorbidae | *Bulinus natalensis* | 0 | 0 | 0 | 0 | 0 | 3 | 0 | 0 |
| Planorbidae | *Bulinus tropicus* | 0 | 0 | 0 | 0 | 0 | 8 | 17 | 0 |
| Planorbidae | *Gyraulus costulatus* | 0 | 0 | 0 | 0 | 2 | 0 | 0 | 0 |
| Platycnemidae | *Allochemis leucosticta* | 7 | 0 | 0 | 0 | 0 | 0 | 0 | 0 |
| Pleidae | *Plea* sp. | 0 | 0 | 0 | 0 | 1 | 0 | 0 | 3 |
| Polymitarcyidae | *Ephoron* sp. | 1 | 0 | 0 | 0 | 0 | 0 | 0 | 0 |
| Psychodidae | *Clogmia albopunctata* | 0 | 0 | 7 | 0 | 0 | 0 | 0 | 0 |
| Scarabaeidae | *Rhyssemus* sp. | 0 | 2 | 0 | 0 | 0 | 0 | 0 | 0 |
| Simulidae | *Simulium* sp. | 2 | 0 | 0 | 0 | 0 | 0 | 0 | 0 |
| Spercheidae | *Spercheus* sp. | 0 | 0 | 0 | 0 | 0 | 2 | 0 | 0 |
| Syrphidae | Syrphidae | 0 | 0 | 0 | 0 | 1 | 0 | 0 | 0 |
| Tabanidae | Tabanidae | 1 | 0 | 0 | 0 | 0 | 0 | 0 | 0 |
| Tabanidae | *Tabanus* sp. | 0 | 0 | 9 | 1 | 0 | 0 | 0 | 0 |
| Tanypodinae | Tanypodinae | 0 | 0 | 49 | 263 | 0 | 96 | 45 | 0 |
| Tetragnathidae | *Tetragnatha* sp. | 0 | 0 | 17 | 0 | 0 | 0 | 0 | 0 |
| Thiaridae | *Melanoides tuberculata* | 0 | 0 | 0 | 1 | 0 | 0 | 0 | 0 |
| Thiaridae | *Tarebia granifera* | 0 | 0 | 0 | 28 | 0 | 0 | 0 | 0 |
| Tipulidae | *Limonia* sp. | 0 | 0 | 1 | 0 | 0 | 0 | 0 | 0 |
| Tipulidae | *Tipula* sp. | 0 | 0 | 12 | 0 | 0 | 0 | 0 | 0 |
| Trichorythidae | *Tricorythus* sp. | 1 | 0 | 0 | 0 | 1 | 0 | 0 | 0 |
| Unionicolidae | Unionicolidae | 0 | 2 | 0 | 0 | 0 | 0 | 0 | 0 |
| Unionicollidae | *Neumania* sp. | 0 | 0 | 24 | 0 | 0 | 0 | 0 | 0 |
| Veliidae | *Angila* sp. | 0 | 0 | 1 | 0 | 0 | 0 | 0 | 0 |
| Veliidae | *Mesovelia* sp. | 0 | 0 | 31 | 19 | 0 | 1 | 1 | 2 |
| Veliidae | *Rhagovelia* sp. | 165 | 0 | 3 | 0 | 0 | 0 | 0 | 0 |

Table S3: The percentage contribution (%) of each of the aquatic invertebrate traits collected during the present study in the Usuthu River (US 18), Lake Shokwe during the low flow (Sh LF) and high flow (Sh HF) and Lake Nyamithi (Nyamithi 18) as well as the Phongolo River (Ph 16), its associated floodplain lakes (FL 1 and FL 4) and Lake Nyamithi (Nyamithi 17) sampled by Dube et al., (2017), de Necker (2019) and de Necker et al. (2021).

| **Sites** | **Us 18** | **Sh LF** | **Sh HF** | **Nya 18** | **Ph 16** | **FL 1** | **FL 4** | **Nya 17** |
| --- | --- | --- | --- | --- | --- | --- | --- | --- |
| **Gills** | 62 | 0 | 18 | 20 | 53 | 48 | 24 | 29 |
| **Tegument/Cutaneous** | 0 | 0 | 1 | 0 | 17 | 0 | 0 | 1 |
| **Aerial: spiracle** | 36 | 89 | 65 | 47 | 6 | 27 | 35 | 31 |
| **Aerial/vegetation** | 0 | 0 | 1 | 0 | 5 | 0 | 1 | 0 |
| **Plastron** | 1 | 4 | 6 | 3 | 5 | 11 | 26 | 24 |
| **Aerial: lungs** | 0 | 0 | 0 | 0 | 0 | 0 | 0 | 0 |
| **unknown respiration** | 2 | 6 | 9 | 29 | 14 | 13 | 13 | 15 |
| **Gravel, sand and mud** | 20 | 0 | 0 | 1 | 7 | 3 | 2 | 10 |
| **Mud** | 0 | 0 | 2 | 7 | 10 | 1 | 8 | 11 |
| **Stones** | 8 | 1 | 1 | 0 | 10 | 17 | 1 | 1 |
| **Indifferent habitat** | 33 | 0 | 15 | 12 | 26 | 23 | 11 | 6 |
| **Vegetation** | 6 | 6 | 14 | 11 | 22 | 36 | 55 | 30 |
| **Free living** | 32 | 86 | 59 | 40 | 4 | 7 | 12 | 28 |
| **Sand** | 0 | 0 | 0 | 0 | 0 | 0 | 0 | 0 |
| **unknown habitat** | 2 | 6 | 9 | 29 | 21 | 14 | 12 | 14 |
| **Pools** | 24 | 7 | 19 | 13 | 45 | 34 | 62 | 54 |
| **Indifferent hydraulic** | 59 | 85 | 67 | 45 | 34 | 6 | 2 | 21 |
| **Bottom dwelling** | 0 | 0 | 0 | 0 | 12 | 0 | 0 | 0 |
| **Riffles** | 3 | 0 | 0 | 0 | 2 | 4 | 9 | 0 |
| **Runs** | 0 | 0 | 0 | 0 | 0 | 15 | 0 | 0 |
| **Rapid** | 1 | 0 | 0 | 0 | 0 | 0 | 0 | 0 |
| **Temporary pools** | 0 | 2 | 2 | 2 | 0 | 4 | 2 | 10 |
| **Unknown hydraulic preference** | 13 | 6 | 12 | 40 | 7 | 37 | 24 | 15 |
| **Aquatic active** | 58 | 2 | 17 | 14 | 55 | 50 | 40 | 38 |
| **Aerial active** | 23 | 91 | 52 | 48 | 9 | 25 | 30 | 36 |
| **Aquatic passive** | 1 | 0 | 0 | 0 | 0 | 2 | 0 | 1 |
| **Unknown dispersal** | 18 | 8 | 30 | 38 | 36 | 23 | 30 | 24 |
| **Predator** | 38 | 90 | 73 | 60 | 36 | 58 | 68 | 58 |
| **Scraper** | 1 | 4 | 6 | 4 | 5 | 26 | 20 | 11 |
| **Grazer** | 3 | 0 | 0 | 7 | 0 | 0 | 0 | 1 |
| **Filter feeder** | 0 | 0 | 1 | 0 | 19 | 0 | 0 | 0 |
| **Deposit feeder 1** | 33 | 0 | 11 | 24 | 26 | 1 | 1 | 20 |
| **Deposit feeder 2** | 13 | 0 | 1 | 0 | 6 | 7 | 10 | 10 |
| **Deposit feeder 3** | 11 | 0 | 0 | 0 | 0 | 0 | 0 | 0 |
| **Omnivore** | 0 | 0 | 0 | 0 | 0 | 0 | 0 | 0 |
| **Shredder** | 0 | 0 | 1 | 0 | 0 | 0 | 0 | 0 |
| **Unknown FFG** | 2 | 6 | 8 | 5 | 8 | 8 | 1 | 0 |
| **Highly tolerant** | 4 | 91 | 69 | 62 | 40 | 77 | 51 | 33 |
| **Moderately tolerant** | 78 | 5 | 24 | 15 | 23 | 18 | 41 | 40 |
| **Moderately sensitive** | 14 | 1 | 2 | 0 | 5 | 4 | 6 | 14 |
| **Highly sensitive** | 3 | 0 | 1 | 0 | 0 | 0 | 0 | 0 |
| **Unknown sensitivity** | 2 | 3 | 4 | 23 | 31 | 2 | 2 | 14 |

Table S4: Water quality variables collected during the present study in the Usuthu River (US 18), Lake Shokwe during the low flow (Sh LF) and high flow (Sh HF) and Lake Nyamithi (Nyamithi 18) as well as the Phongolo River (Ph 16), its associated floodplain lakes (FL 1 and FL 4) and Lake Nyamithi (Nyamithi 17) sampled by Dube et al., (2017), de Necker (2019) and de Necker et al. (2021). – indicates not measured.

| **Sites** | **pH** | **Turbidity (mg/L)** | **Sulphates (mg/L)** | **Orthophosphates (mg/L)** | **Nitrites (mg/L)** | **Nitrates (mg/L)** | **Chlorides (mg/L)** | **Ammonium (mg/L)** | **COD (mg/L)** | **TDS (mg/L)** | **TH (mg/L)** |
| --- | --- | --- | --- | --- | --- | --- | --- | --- | --- | --- | --- |
| **US 18** | 8.4 | 25 | 43 | 0.07 | 0.13 | 3.1 | 30 | 0.43 | 6.3 | 239 | 14 |
| **Sh HF** | 7.6 | 21 | 49 | 0.07 | 0.04 | 1.8 | 24 | 0.29 | 14 | 187 | 39 |
| **Sh LF** | 7.7 | 52 | 105 | 0.14 | 0.05 | 3.7 | 26 | 0.08 | 15 | 220 | 109 |
| **Nya 18** | 8.5 | 39 | 156 | 0.36 | 0.04 | 1.8 | 237 | 0.43 | 37 | 1630 | 333 |
| **Ph 16** | 8.3 | 83 | 56 | 0.21 | 0.03 | 1.4 | 98 | 0.15 | 4.2 | 494 | 66 |
| **FL 1** | 7.08 | – | 50 | 0.45 | 0.1 | 14.9 | 46 | 0.58 | – | – | 40.38 |
| **FL 4** | 7.69 | – | 53 | 0.55 | 0.12 | 16.85 | 73 | 0.6 | – | – | 28.53 |
| **Nya 17** | 8 | 61 | 1197 | 0.23 | 0.11 | 4 | 2277 | 0.18 | 41 | 7397 | 389 |

Table S5: Percentage dissimilarities contribution of the 28 aquatic macroinvertebrate taxa that contributed ≥1% to differences between the Usuthu River and Lake Nyamithi (Nya 17 and Nya 18) calculated by Similarity of percentage analysis (SIMPER). * indicates taxa present only in the Usuthu River; ^§^ indicates taxa present only Lake Nyamithi.

| Site | Overall dissimilarity (%) | Species | Average dissimilarities (%) | Percentage contribution (%) | Cumulative contribution (%) |
| --- | --- | --- | --- | --- | --- |
| Usuthu vs Lake Nyamithi | **82.16%** | Baetidae | 6.18 | 7.53 | 7.53 |
|  |  | **^§^**Ostracoda | 5.69 | 6.93 | 14.45 |
|  |  | ******Rhagovelia* sp. | 5.39 | 6.56 | 21.01 |
|  |  | *Micronecta* sp. | 5.01 | 6.09 | 27.1 |
|  |  | ******Aulonogyrus* sp. | 4.56 | 5.55 | 32.65 |
|  |  | *Caenis* sp. | 3.1 | 3.77 | 36.42 |
|  |  | **^§^**Tanypodinae | 2.72 | 3.31 | 39.73 |
|  |  | *Caridina nilotica* | 2.67 | 3.25 | 42.98 |
|  |  | **^§^***Bezzia* sp. | 2.58 | 3.14 | 46.12 |
|  |  | *Anisops* sp. | 2.35 | 2.86 | 48.99 |
|  |  | ******Afrocaenis* sp. | 2.34 | 2.84 | 51.83 |
|  |  | **^§^***Hyphydrus* sp. | 2.25 | 2.74 | 54.58 |
|  |  | ******Afronurus* sp. | 2.05 | 2.5 | 57.08 |
|  |  | Oligochaeta | 2.02 | 2.45 | 59.53 |
|  |  | *Berosus* sp. | 1.85 | 2.25 | 61.78 |
|  |  | *Enithares* sp. | 1.35 | 1.64 | 63.42 |
|  |  | ******Nilus margartatus* | 1.33 | 1.61 | 65.04 |
|  |  | **^§^***Brachythemis leucosticta* | 1.31 | 1.59 | 66.63 |
|  |  | **^§^**Chironominae | 1.29 | 1.58 | 68.21 |
|  |  | **^§^***Nychia limpida* | 1.26 | 1.53 | 69.74 |
|  |  | ******Allocnemis leucosticta* | 1.11 | 1.35 | 71.09 |
|  |  | ******Hydropsyche* sp. | 1.11 | 1.35 | 72.44 |
|  |  | **^§^***Mesovelia* sp. | 1.09 | 1.32 | 73.77 |
|  |  | **^§^***Agraptocorixa* sp. | 1.05 | 1.27 | 75.04 |
|  |  | **^§^***Sigara* sp. | 0.98 | 1.19 | 76.23 |
|  |  | **^§^***Rhagadotarsus hutchinsonii* | 0.93 | 1.13 | 77.36 |
|  |  | **^§^***Tarebia granifera* | 0.89 | 1.08 | 78.44 |
|  |  | *Appasus* sp. | 0.87 | 1.06 | 79.5 |

Table S6: Percentage dissimilarities contribution of the 34 aquatic macroinvertebrate taxa that contributed ≥1% to differences between the Phongolo River and Lake Nyamithi (Nya 17 and Nya 18) calculated by Similarity of percentage analysis (SIMPER). * indicates taxa present only in the Phongolo River; ^§^ indicates taxa present only in Flake Nyamithi.

| Site | Overall dissimilarity (%) | Species | Average dissimilarities (%) | Percentage contribution (%) | Cumulative contribution (%) |
| --- | --- | --- | --- | --- | --- |
| Phongolo vs Lake Nyamithi | **82.49%** | **^§^**Ostracoda | 6.31 | 7.65 | 7.65 |
|  |  | *Micronecta* sp. | 5.83 | 7.07 | 14.72 |
|  |  | ******Thermocyclops* sp. | 3.23 | 3.92 | 18.64 |
|  |  | **^§^***Berosus* sp. | 3.02 | 3.66 | 22.3 |
|  |  | **^§^**Tanypodinae | 2.97 | 3.6 | 25.89 |
|  |  | **^§^***Bezzia* sp. | 2.84 | 3.45 | 29.34 |
|  |  | Chironominae | 2.82 | 3.42 | 32.76 |
|  |  | **^§^***Hyphydrus* sp. | 2.54 | 3.08 | 35.85 |
|  |  | *****Chydoridae | 2.45 | 2.97 | 38.82 |
|  |  | **^§^**Oligochaeta | 2 | 2.42 | 41.24 |
|  |  | ******Tetrathemis polleni* | 1.94 | 2.36 | 43.59 |
|  |  | *Anisops* sp. | 1.78 | 2.16 | 45.76 |
|  |  | ******Physella acuta* | 1.56 | 1.9 | 47.65 |
|  |  | ******Simocephalus serrulatus* | 1.56 | 1.9 | 49.55 |
|  |  | **^§^***Brachythemis leucosticta* | 1.5 | 1.82 | 51.37 |
|  |  | *Culex* sp. | 1.45 | 1.76 | 53.13 |
|  |  | **^§^***Nychia limpida* | 1.44 | 1.75 | 54.88 |
|  |  | **^§^***Enithares* sp. | 1.38 | 1.67 | 56.55 |
|  |  | *Pseudagrion* sp. | 1.37 | 1.66 | 58.21 |
|  |  | Baetidae | 1.28 | 1.55 | 59.76 |
|  |  | Cyprididae | 1.25 | 1.51 | 61.27 |
|  |  | *****Darwinulidae | 1.25 | 1.51 | 62.79 |
|  |  | *Corbicula fluminalis* | 1.23 | 1.49 | 64.28 |
|  |  | **^§^***Laccocoris* sp. | 1.22 | 1.48 | 65.77 |
|  |  | **^§^***Mesovelia* sp. | 1.21 | 1.46 | 67.23 |
|  |  | **^§^***Appasus* sp. | 1.16 | 1.4 | 68.63 |
|  |  | **^§^***Agraptocorixa* sp. | 1.14 | 1.39 | 70.01 |
|  |  | **^§^***Sigara* sp. | 1.12 | 1.36 | 71.37 |
|  |  | ******Cloeon* & *Procloeon* | 1.05 | 1.28 | 72.65 |
|  |  | **^§^***Rhagadotarsus hutchinsonii* | 1.02 | 1.23 | 73.88 |
|  |  | **^§^***Tarebia granifera* | 0.97 | 1.17 | 75.06 |
|  |  | *Caridina nilotica* | 0.95 | 1.15 | 76.21 |
|  |  | ******Anax* sp. | 0.94 | 1.14 | 77.35 |
|  |  | *Laccobius* sp. | 0.84 | 1.02 | 78.37 |

Supplementary figures


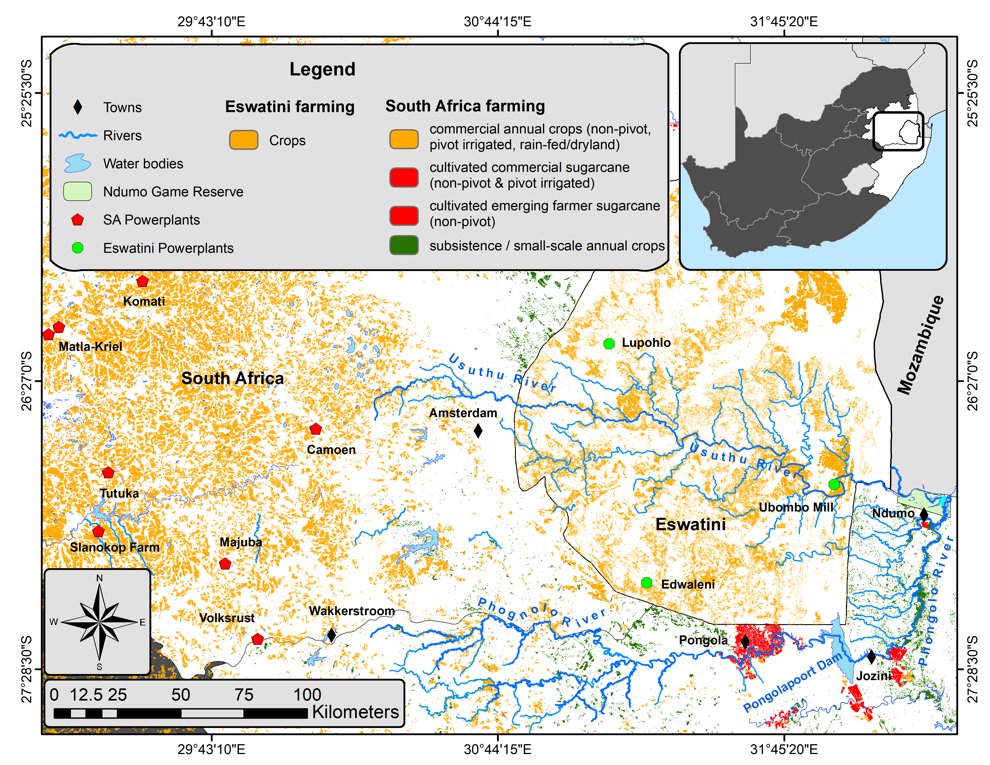


Figure S1: Map of the Usuthu – Phongolo rivers catchment area that includes both the Phongolo and Usuthu rivers and their associated impacts in the upper catchments.


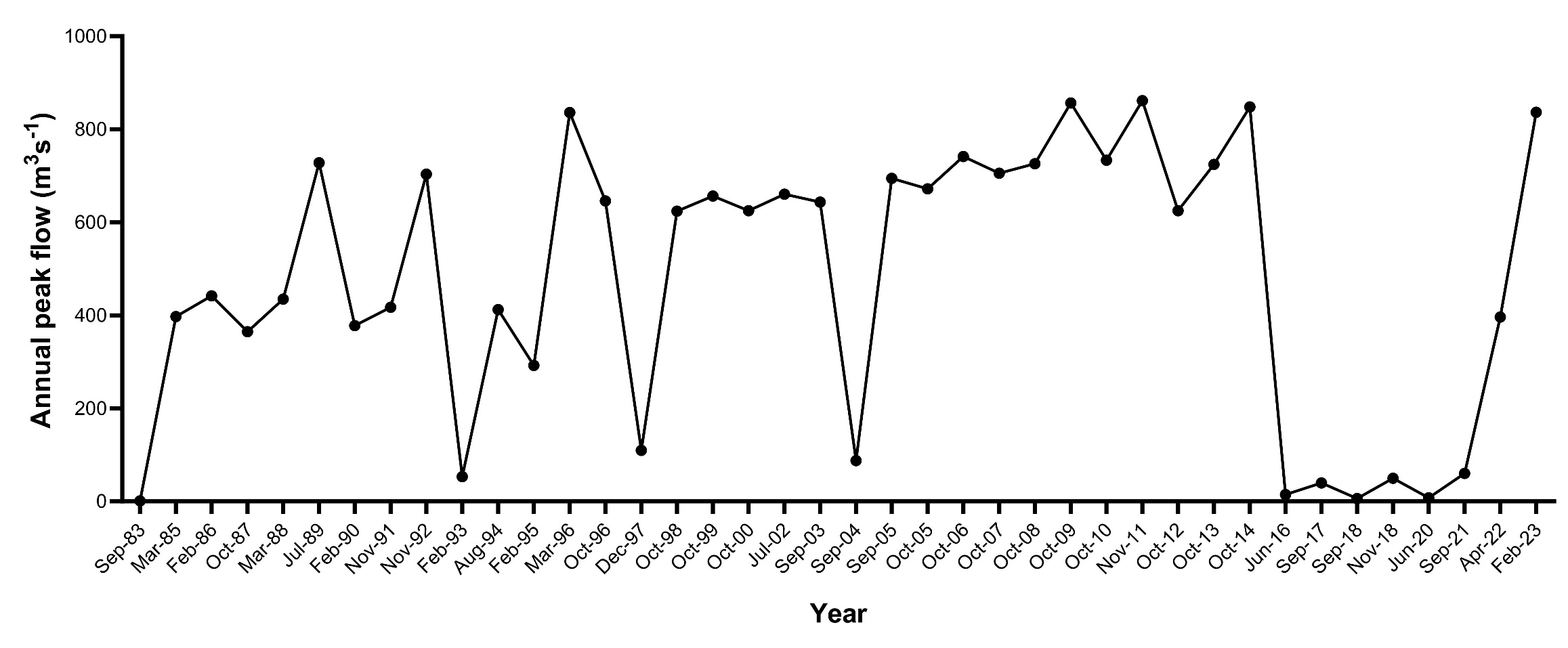


Figure S2: Annual peak flows of the Lower Phongolo River downstream of the Pongolapoort Dam measured from 1983 to 2023. (available from https://www.dwaf.gov.za/Hydrology/Verified/hymain.aspx. Accessed 18 September 2023).


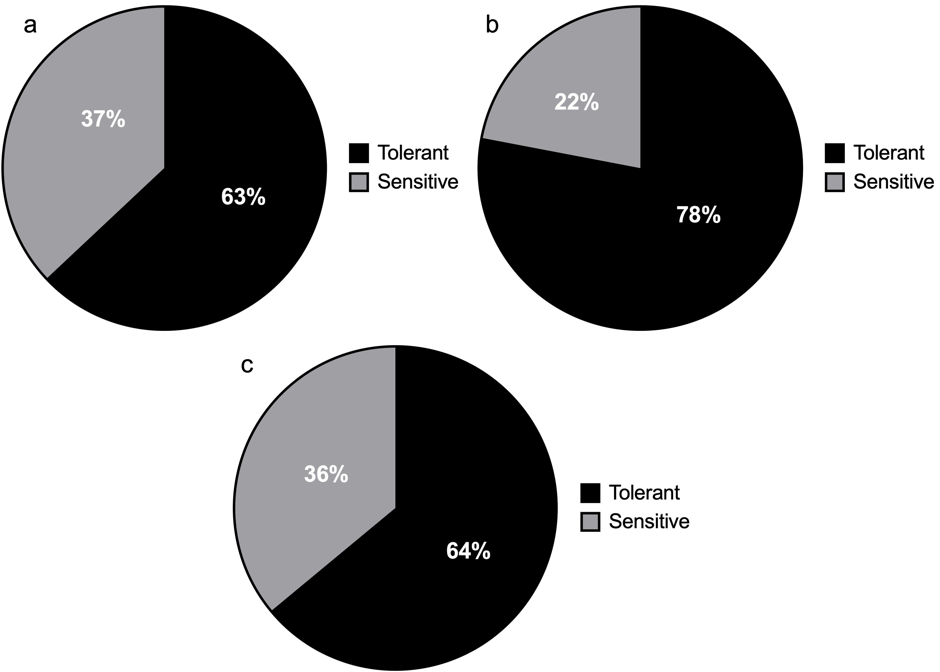


Figure S3: Pie charts indicating percentage sensitive and tolerant aquatic macroinvertebrate family scores of different surveys on the Phongolo River during 2012-2013 (a) collected by Smit et al., 2016 (b) collected by de Necker, 2019 and the present study on the Usuthu River (c).
